# Nanoporous Capillary Gripper for Ultragentle Micro‐Object Manipulation

**DOI:** 10.1002/advs.202508338

**Published:** 2025-06-25

**Authors:** Seong Jae Kim, Taehoon Kim, Hyun Jun Ryu, Ji‐hun Jeong, A. John Hart, Sanha Kim

**Affiliations:** ^1^ Department of Mechanical Engineering, Korea Advanced Institute of Science and Technology (KAIST) Daejeon 34141 Republic of Korea; ^2^ Department of Mechanical Engineering Massachusetts Institute of Technology (MIT) Cambridge MA 02139 USA

**Keywords:** adhesion, capillary force, pick‐and‐place, vertically‐aligned carbon nanotubes

## Abstract

Surfaces become “sticky” at the micro/nano length scale as the gravitational force is no longer effective. Ultragentle, high‐contrast switching of interfacial adhesion is the key to reliable small‐scale object manipulation. Here, a novel approach is presented for surface adhesion control utilizing a liquid‐permeable nanoporous surface, which can switch from off‐state adhesion (< 0.002 kPa) to on‐state attraction (0.8 kPa) without preload. The surface of the gripper is composed of vertically aligned composite nanowires with an average diameter of 79 nm. When a liquid is injected into the nanoporous membrane, capillary adhesion occurs, allowing the object to be picked up. As the liquid evaporates, the object can be released by extremely sparse contact. The off‐state adhesion of a millimeter‐scale gripper is even lower than the gravitational force of thin polymer films (0.18 mN cm^−2^), enabling the solid‐contactless release of lightweight materials. We characterize and model the mechanism across length scales and provide pick‐and‐place demonstrations including LED chips, micro‐architected materials, and thin‐film electronics.

## Graphical abstract

Microscale pick‐and‐place requires low‐pressure handling and controllable adhesion due to the increased fragility and reduced size of components. A capillary gripper with nanoporous surface enables switchable capillary adhesion, achieving ultralow “off‐state” stickiness for reliable micro‐scale pick‐and‐place of delicate objects. Liquid injection allows pickup; evaporation enables solid‐contactless release, demonstrated with LED chips, micro‐architected materials, and thin‐film electronics.

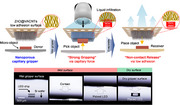

## 1 Introduction

Accurately moving and positioning milli‐ and micro‐scale objects is significant to diverse fields, including electronics,^[^ ^1^ ^]^ biotechnology,^[^ ^2^ ^]^ and photonics.^[^ ^3^ ^]^ For instance, the process of placing micro‐LEDs has recently garnered substantial industrial attention owing to the lower power consumption of micro‐LED displays.^[^ ^4^ ^]^ Additionally, with advancements in stretchable electronics and 3D integrated circuits, transfer printing and heterogeneous integration have found increasing applications.^[^ ^5^, ^6^, ^7^, ^8^ ^]^ However, transfer printing of discrete objects becomes much more difficult to control as the size scale of objects drops.

Adhesion is a complicated phenomenon that involves multiple mechanisms working together. In pick‐and‐place processes, handling capability often relies on the gravitational force and the van der Waals force. At the macroscale, intermolecular forces are much smaller compared to the gravitational force. However, gravitational force rapidly diminishes as the object size decreases.^[^ ^9^ ^]^ Consequently, the van der Waals force gains more prominence at the submillimeter scale, making it difficult to reliably transfer microscopic objects.

As the demands for micro‐ and nanoscale transfer printing have evolved, printing with low contact pressure is necessary to integrate ultrathin and micro‐architected elements that are sensitive to applied pressure.^[^ ^10^, ^11^, ^12^, ^13^ ^]^ In particular, the thickness of dies employed in the advanced packaging and heterogeneous integration continuously decreases to match the industrial needs of high‐performance computing,^[^ ^8^ ^]^ which means that the structures become more fragile. To satisfy this requirement, the adhesion of the gripping tool should be controlled with minimal applied pressure. This demand cannot be met with conventional stamp‐based transfer equipment, where contact pressure significantly contributes to the operating mechanism.

To address the challenges associated with the placement of small objects, various techniques with active adhesion control capabilities have been studied. Strategies considered include active modulation of the contact area,^[^ ^14^ ^]^ kinetic control of adhesion of viscoelastic elastomers,^[^ ^15^ ^]^ and use of electrostatic,^[^ ^16^, ^17^, ^18^ ^]^ magnetic,^[^ ^19^ ^]^ and capillary interactions.^[^ ^20^ ^]^ Among these, capillary forces do not require any preload to apply attractive pressure and therefore allow for the transfer of delicate objects.^[^ ^21^ ^]^ Yet, a further significant challenge is the high adhesive force between the object and the substrate, which necessitates an approach with a high adhesion contrast: strong adhesion to pick up the object, with minimum preload, and maximally weak adhesion to place the object without causing damage.

**Figure 1:**
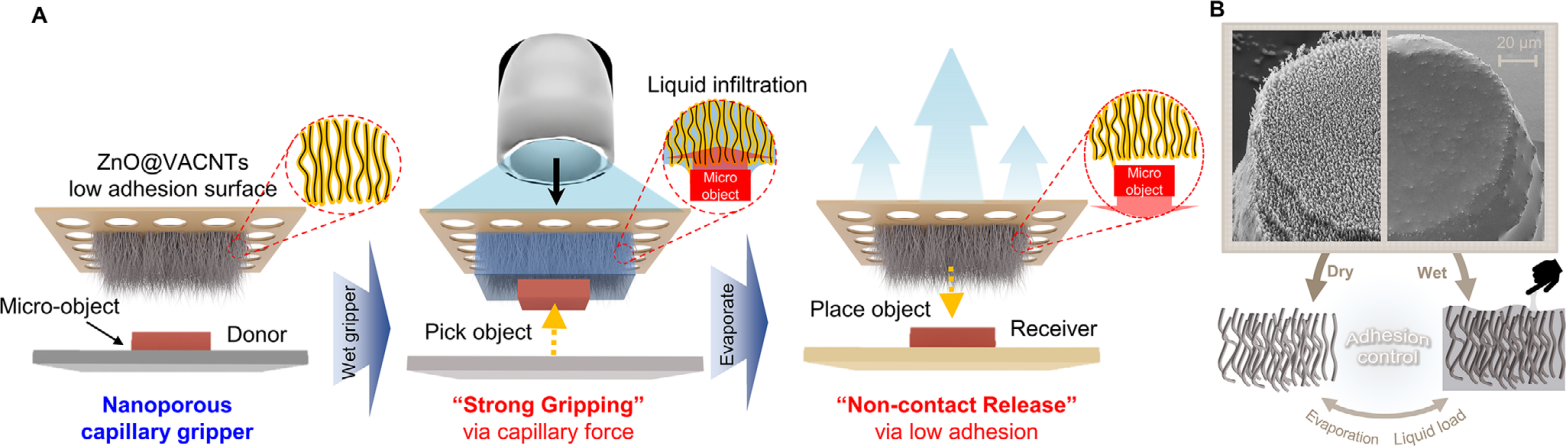
Nanoporous capillary gripper for small object pick and place. A) Sequential depiction of the process flow and working principle. Upon liquid introduction, capillary adhesion enables object pickup, and as the liquid evaporates, the reduced adhesion allows for non‐contact placement, B) Representative surface morphology before and after liquid wetting.

We present a novel capillary gripper for ultragentle manipulation of sub‐millimeter objects. The surface of the gripper comprises vertically‐aligned composite nanowire arrays, that enable liquid infiltration and evaporation without changes in shape (**Figure** **1**A). Owing to the extremely low contact area of the nanoporous surface and the preload‐independent nature of capillary adhesion, the gripper exhibits very low surface adhesion in the off (dry) state. For picking, the wet surface creates an attractive pressure due to its capillarity contribution (Figure 1B). Adhesion characterization across multiple scales and contact mechanical modeling revealed the influence of various operating parameters such as liquid type, volume, and preload. Also, we theoretically and experimentally study the usage of external Joule heating to improve the switching speed of the capillary gripper. For demonstrations of small object manipulation, LED chips, micro‐architected materials, and thin‐film electronics were successfully transferred with an extremely small contact pressure.

## 2 Results and Discussion

### 2.1 Engineered Nanoporous Surface of the Capillary Gripper

**Figure 2:**
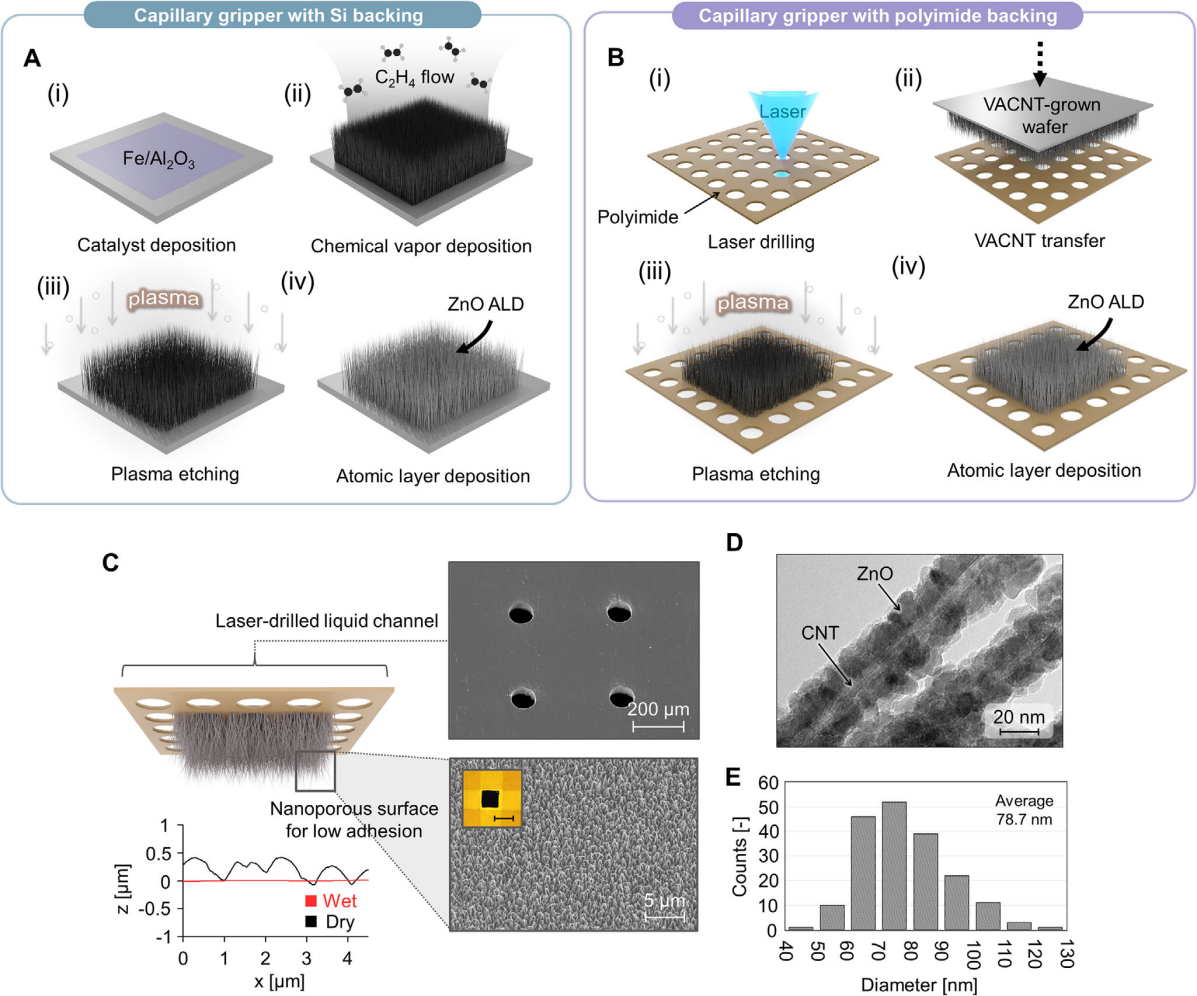
Structure and fabrication process of a nanoporous capillary gripper. Stepwise schematic of the fabrication process: A) For capillary grippers with Si backing, the catalyst was deposited on a Si wafer followed by CVD growth of VACNTs, plasma etching, and ALD deposition of ZnO, and B) for those with polyimide backing, the CVD‐grown VACNTs were dry transferred onto laser‐drilled polyimide film, followed by plasma etching and ZnO‐ALD. (C) SEM images of the capillary gripper with polyimide backing taken from top and bottom, and the surface profiles of the top nanoporous surface under wet and dry conditions. (Inset: Optical image of the as‐fabricated gripper. Scale bar: 5 mm.) D) TEM image of the ZnO‐coated CNTs and (E) histogram showing the diameter distribution of the nanowires after ZnO‐ALD.

The capillary gripper uses a nanoporous surface which is reversibly wetted and dried to modulate its adhesion during pick and place operations, resulting in a high adhesion contrast and extremely low off‐state adhesion. The capillary gripper could be fabricated in two different ways. First, a Si wafer or a hole‐drilled polyimide film was selected as the back substrate of the gripper. When using a Si wafer as the back substrate, the fabrication process was proceeded in four steps: (i) catalyst patterning, (ii) synthesis of vertically‐aligned carbon nanotube (VACNT), (iii) plasma etching, and (iv) atomic layer deposition (ALD), **Figure** **2**A. In the case of the polyimide substrate, the transfer step to a hole‐drilled polyimide adhesive was included, and the rest of the process was the same, Figure 2B. We patterned the polyimide films using a femtosecond laser to provide liquid channels with a diameter of 85 µm (Figure 2C; Figure S1, Supporting Information). By using these hole arrays, the liquid could be applied from the backside of the gripper before the picking step, and the low surface tension allowed the liquid (e.g., ethanol) to spread over the entire surface. The key difference between the two designs (Si and polyimide backing) is the gripper wetting method. Although the polyimide backing requires an additional fabrication step, it enables continuous liquid supply from the back, preventing unnecessary delays during the pick‐and‐place process. Both designs employ the same ZnO‐coated VACNT structure, thereby achieving low adhesion properties. For example, light object (<0.6 mg) can be separated without solid contact, regardless of whether the gripper has a Si backing or a polyimide backing (Movies S1 and S2, Supporting Information).

To establish the nanoporous surface of the gripper, we synthesized VACNT layers via chemical vapor deposition (CVD) from catalyst‐deposited wafers. VACNTs have a well‐controlled, oriented nanoporous structure; for example, if the catalyst layer is patterned using photoresist, a complicated VACNT surface of a desired design can be produced.^[^ ^22^ ^]^ As‐synthesized CNTs exhibited ≈10 nm in diameter. Additionally, the height of the VACNTs was controlled over a wide range from 10 µm to 1 mm by changing the hydrocarbon precursor exposure time during the CVD process (Figure S2, Supporting Information).

After the CVD growth, we further engineered the VACNTs, comprising dry plasma etching followed by ALD, which increased the porosity at the top surface and enhanced the mechanical robustness of the nanoporous structure, respectively. Plasma treatment of VACNTs effectively removed the entangled CNT clusters on the top surface,^[^ ^23^ ^]^ contributing to the formation of a low contact area (Figure S3, Supporting Information). ALD could conformally and precisely coat the surface of nanostructured surfaces since its vapor‐phase precursor could be diffused even inside the nanoporous network of the VACNTs.^[^ ^16^ ^]^ To reinforce the VACNT network^[^ ^24^, ^25^ ^]^ and improve its stability upon wetting and drying, ALD of ZnO on the CNTs was performed. By TEM imaging, we confirmed that an ultrathin ZnO layer was successfully deposited on CNTs, Figure 2D. The average diameter of the nanowires was ≈78.7 nm according to the SEM images (Figure 2E; Figure S4, Supporting Information). Such a uniform yet thin ceramic coating not only reinforced individual nanotubes but also created connections between adjacent CNTs, therefore providing enough mechanical resistance during liquid infiltration. The ZnO‐coated VACNTs retained their shape after evaporation, verifying that the nanoporous structure was strong enough to prevent elastocapillary densification (Movie S3 and Figure S5, Supporting Information). We also conducted nanoscopic and macroscopic indentation experiments, which showed that ZnO‐ALD significantly increased the contact stiffness and reduced the residual displacement after indentation, thereby enhancing the robustness of the structure (Figures S6–S11, Supporting Information).

The fabricated nanoporous gripper in dry and wet states appeared as shown in Figure 2C. The surface of ZnO‐coated VACNT was composed of numerous nanowires aligned in the out‐of‐plane direction. In the dry state, the surface was macroscopically flat but microscopically rough. In wet‐state, however, the liquid covered the aligned nanowires and therefore the surface became smooth and flat. For example, tetradecane was infiltrated inside the nanoporous layer, and the resulting average roughness was ≈6.1 nm, Figure S12 (Supporting Information).

### 2.2 Adhesion Properties of the Wet, Nanoporous Surface

**Figure 3:**
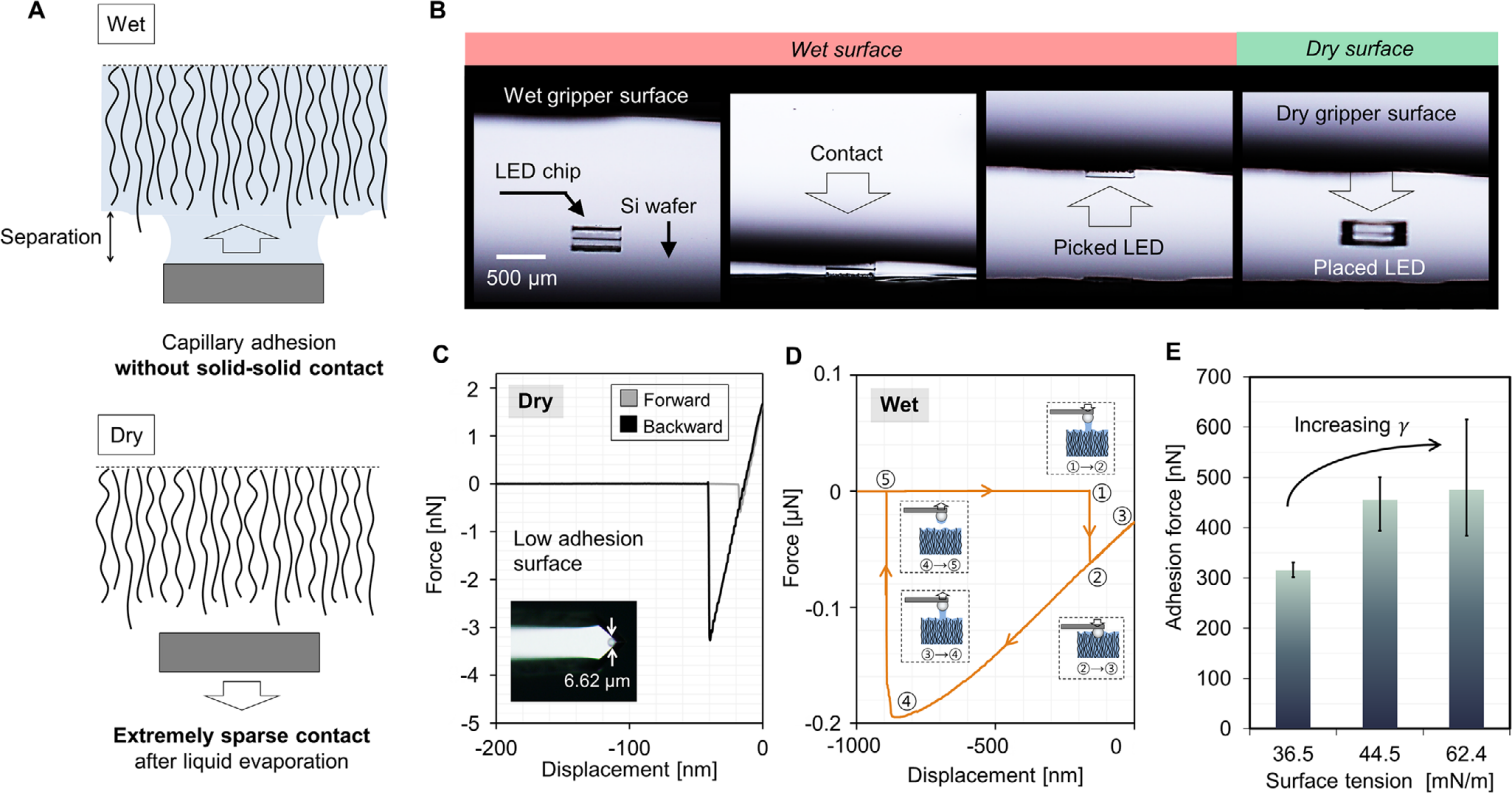
Adhesion characteristics of wet and dry switchable nanoporous surfaces. A) Schematics showing the key mechanism enabling ultragentle manipulation via high‐contrast adhesion switching between wet and dry states. B) Side‐view images of the pick‐and‐place operation of a miniature LED chip (0.22 mg, 600 × 600 µm^2^). Here, the nanoporous gripper surface is wetted by ethanol for picking and evaporated for placing. Microscopic adhesion measurements via AFM on C) a dry gripper surface exhibiting only 2.6 nN pull‐off force and D) a tetradecane‐wetted surface with an enhanced pull‐off force of 195 nN. E) Comparison of adhesion forces on wetted nanoporous gripper surface according to different surface tension controlled by the glycerol–propylene glycol mixtures of varying mole fractions.

The wet, nanoporous surfaces exhibit (i) preload‐independent adhesion and (ii) high adhesion contrast between wet and dry states. The capillary force is generated by the Laplace pressure due to the distortion of the capillary bridge formed between two surfaces and the surface tension force from the meniscus. For the wet nanoporous surface, therefore, direct contact is not necessary to create attractive pressure. The surface can be switched into a dry surface as liquid evaporates, then only the extremely sparse solid‐solid contact remains, **Figure** **3**A.

As the liquid evaporates with the object held on the gripper, the negligible contact between the stamp and the target in the dry state enables “solid‐contactless” placing of lightweight objects. For instance, Figure 3B and Movie S4 (Supporting Information) show the release of a sub‐millimeter‐scale LED chip (0.22 mg, 600 × 600 µm^2^) without contact. Interestingly, we confirmed that noncontact placement was even possible with an extremely light polymer (polyimide) film (0.33 mg with a contact area of ≈1.8 mm^2^), Movie S2 (Supporting Information). The result implies that the nominal adhesive pressure was smaller than 1.8 Pa, supporting the capability of solid‐contactless placement for integration of thin electronics.

To quantitatively understand the adhesion contrast of the nanoporous gripper, we performed microscopic single‐asperity adhesion testing (a probe diameter of 6.62 µm) using AFM. When the tip contacted the dry surface, the adhesion strength was as low as 2.6 nN, Figure 3C. Despite repeated measurements with a preload of ≈10 nN, the adhesion did not increase beyond 15 nN (Figure S13, Supporting Information). In contrast, the adhesion strength significantly increased when the liquid was supplied to the gripper, Figure 3D. Tetradecane (C_14_H_30_), a nonvolatile liquid with dynamic viscosity of ∼2 mPa·s,^[^ ^26^ ^]^ along with comparable surface tension to ethanol (≈22 N m^−1[^ ^27^ ^]^ versus 27 N m^−1[^ ^28^, ^29^ ^]^) was used. The maximum pull‐off force measured against the wetted surface was ≈195 nN, and 75 times higher than the adhesion on a dry surface.

The magnitude of the on‐state adhesion and therefore the pull‐off force depends on the liquid‐air surface tension (*γ*).^[^ ^30^ ^]^ To investigate the effects of liquid selection on capillary adhesion, Figure 3E, binary mixtures of propylene glycol (C_3_H_8_O_2_, γ = 36.2 N m^−1^) and glycerol (C_3_H_8_O_3_, γ = 62.4 N m^−1^) were used because they exhibit low vapor pressures and perfect mutual miscibility.^[^ ^31^, ^32^ ^]^ The represented surface tension values were obtained from experimental data provided by previous literature. We observed a monotonic decrease in the maximum adhesion force, proportional to the mole fraction of propylene glycol. While the adhesion force in the liquid bridge may arise from both capillary force and viscous force,^[^ ^33^, ^34^, ^35^ ^]^ the contribution from dynamic viscosity is expected to be insignificant due to the retraction velocity of 1 µm s^−1^.

### 2.3 Gripping Performance and Mechanics of Nanoporous Capillary Gripper

**Figure 4:**
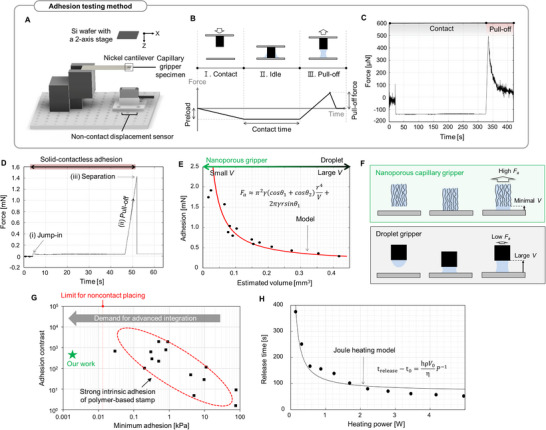
Analysis of gripping performance and the underlying mechanics of the nanoporous capillary gripper. A) Schematic of the custom‐built cantilever‐type adhesion tester. B) Expected contact and pull‐off behavior for capillary adhesion and C) actual measured capillary adhesion with ethanol. D) Measured solid‐contactless adhesion (no preload) from the nanoporous gripper surface with infiltrated ethanol inside the pores. E) Measured capillary adhesion according to the volume of the liquid bridge. F) Working principles for nanoporous gripper surface with small liquid volume, achieving high adhesion force in contrast to the large droplet, resulting in low capillary adhesion. G) A combination of large adhesion switchability and low minimum adhesion achieved by our nanoporous gripper, which contrasts with other existing controllable adhesion strategies (10, 14, 16, 17, 36–46). H) Accelerated release time by liquid evaporation via Joule heating for rapid pick‐and‐place.

To investigate the major process parameters and material design factors that determine the nanoporous capillary gripping performance, a lab‐built adhesion tester was used to measure the microscopic adhesion force, **Figure** **4**A. The adhesion was estimated by measuring the cantilever deflection, which is proportional to the force. Each adhesion testing was conducted in three steps: contact, idle, and pull‐off, Figure 4B, and the pull‐off force was determined as the maximum force in the retraction curve. The contact force before pull‐off, in general referred to as “preload”, is important in most of the contact‐based adhesion, therefore we precisely controlled the preloads in every experiment. A typical force‐displacement curve with a reload using ethanol as the working liquid is acquired as shown in Figure 4C.

The main advantage of our nanoporous capillary gripper, the preload‐insensitivity, was confirmed again via the solid‐contactless adhesion measurement, Figure 4D. For our wet nanoporous surfaces, we could obtain high pull‐off force even when no contact force was applied before the retraction step. For example, we observed strong attractive pressure (≈0.8 kPa for ≈1.8 mm^2^ of contact area) even when there was no solid‐solid contact. In addition, such capillary adhesion strength did not greatly vary according to the preload (Figure S14, Supporting Information). While most other contact‐based adhesion heavily relies on high preloads to achieve strong adhesion, the nanoporous capillary gripper can operate at its full strength without the preload.

As the adhesion of the wet nanoporous surface relies on the formation of the liquid bridge, the adhesive strength may depend on the volume of fluid held in the nanoporous surface. We experimentally measured the adhesion values according to different liquid volumes by changing the evaporation time, Figure 4E. When a small volume of liquid (0.024 mm^3^) is used, the pull‐off force is 6.58 times greater than the case of the largest volume (0.42 mm^3^). To better understand the effect of liquid volume on the gripping force, we compared our experimental results with an analytical model. The capillary adhesion (*F_a_*) of our gripper can be approximated as:^[^ ^30^ ^]^

(1) $$F_{a}\approx 2\pi r\gamma sin\theta_{1}+\pi^{2}\gamma \left(cos\theta_{1}+cos\theta_{2}\right)r^{4}V^{-1}$$

where *r* is the neck radius of the capillary bridge, *θ_1_* and *θ_2_* are the contact angles of the gripper and counterpart object surface, and *V* is the volume of the liquid bridge (details in the supplementary material).

The model estimation and experiment showed a good agreement. In case when the applied liquid volume is large, the first term (surface tension force) in Equation 1 dominates the second term (Laplace pressure). Accordingly, the capillary force is proportional to the diameter of the contact area, which is supported by experimental results (Figure S15, Supporting Information). However, the Laplace pressure increases rapidly as the liquid volume decreases (*F ∝ V^−1^*), indicating that a stronger gripping force can be achieved when operating with a small volume of liquid. Such a working principle is advantageous for using our wet, nanoporous gripper. Compared to a general capillary gripper in which a droplet is wetted on the surface, Figure 4F, a nanoporous capillary gripper can form a meniscus with a very small volume,^[^ ^36^ ^]^ therefore, can obtain strong capillary adhesion. As predicted by the model, contact angle can also contribute to the capillary adhesion force. However, being a dynamic contact angle, it does not solely depend on wettability, and experimental results show that capillary adhesion is not significantly different across various contacting surfaces (e.g., Si, Cu, Au, and Teflon) (Figure S16, Supporting Information).

Although capillary force measurements in Figure 4F were performed at the millimeter scale, previous literature has reported quantitative characterization of capillary forces at smaller scales.^[^ ^37^, ^38^ ^]^ The capillary force scales linearly with the length scale, which suggests that capillary force becomes more predominant over gravitational force at small scales (Figure S17, Supporting Information). Thus, the capillary gripping mechanism can be exploited at much smaller scales, as long as the object is sufficiently larger than the nanowire diameter.

We compare the adhesion tunability of the nanoporous capillary gripper with other existing techniques,^[^ ^10^, ^14^, ^16^, ^17^, ^39^, ^40^, ^41^, ^42^, ^43^, ^44^, ^45^, ^46^, ^47^, ^48^, ^49^ ^]^ Figure 4G. To the best of our knowledge, our approach achieves an unprecedented range of adhesion control along with exceptionally low “off‐state” adhesion (nominal attractive pressure of 1.8 Pa for a 1.8 mm^2^ contact area) and enabling solid‐contactless picking without preload, Table S3 (Supporting Information). Non‐contact placement of a wafer die typically requires a nominal adhesion force lower than 12 Pa, which remains difficult to attain with conventional strategies, thereby demonstrating the advantage of our approach.

Lastly, we show that the pick‐and‐place cycle time can be improved using Joule heating for liquid evaporation. After picking an object with capillary force, we should evaporate the liquid inside the porous structure to diminish the adhesion. By enhancing the evaporation time, we can realize a rapid operation cycle for pick‐and‐place. A film heater with a resistance of 29 Ω was attached to the back of the gripper, and the release time was evaluated under varying heating powers. The release time was measured using an adhesion tester, specifically recording the duration required for the cantilever and the CNT surface to separate under an initial adhesion force of ∼1 mN (Figure S18, Supporting Information). Given that most of the energy provided by external Joule heating is likely consumed as latent heat during the liquid‐to‐vapor phase transition,^[^ ^20^ ^]^ the following relationship can be established.

(2) $$t_{release}-\;t_{0}=\frac{h\rho V_{0}}{\eta }\;P^{-1}$$

where *t_release_* is the time for object release, *t_0_* is the reference time for the heating initiation, *h* is the specific latent heat, *ρ* is the density of liquid, *V_0_* is the initial volume of the liquid, *η* is the efficiency of the heat transfer, and *P* is the heating power. We measured five‐fold faster liquid evaporation with a power of ≈5 W, Figure 4H, compared to ambient conditions. The trend (*t_release_ ∝ P^−1^*) predicted by the model was well aligned with the experimental results.

In addition, the time required for object release can be further reduced by improving the Joule heater design and decreasing the liquid volume. For example, instead of attaching a commercial film heater to the back of the capillary gripper, a Joule heater can be realized by directly depositing 40 nm Pt layer on the back of the polyimide, which resulted in better heat transfer efficiency and enabled object placement in less than 10 s (Figures S19–S22 and Movie S5, Supporting Information).

### 2.4 Nanoscale Contact Mechanics of the Dry Nanoporous Gripper Surface

**Figure 5:**
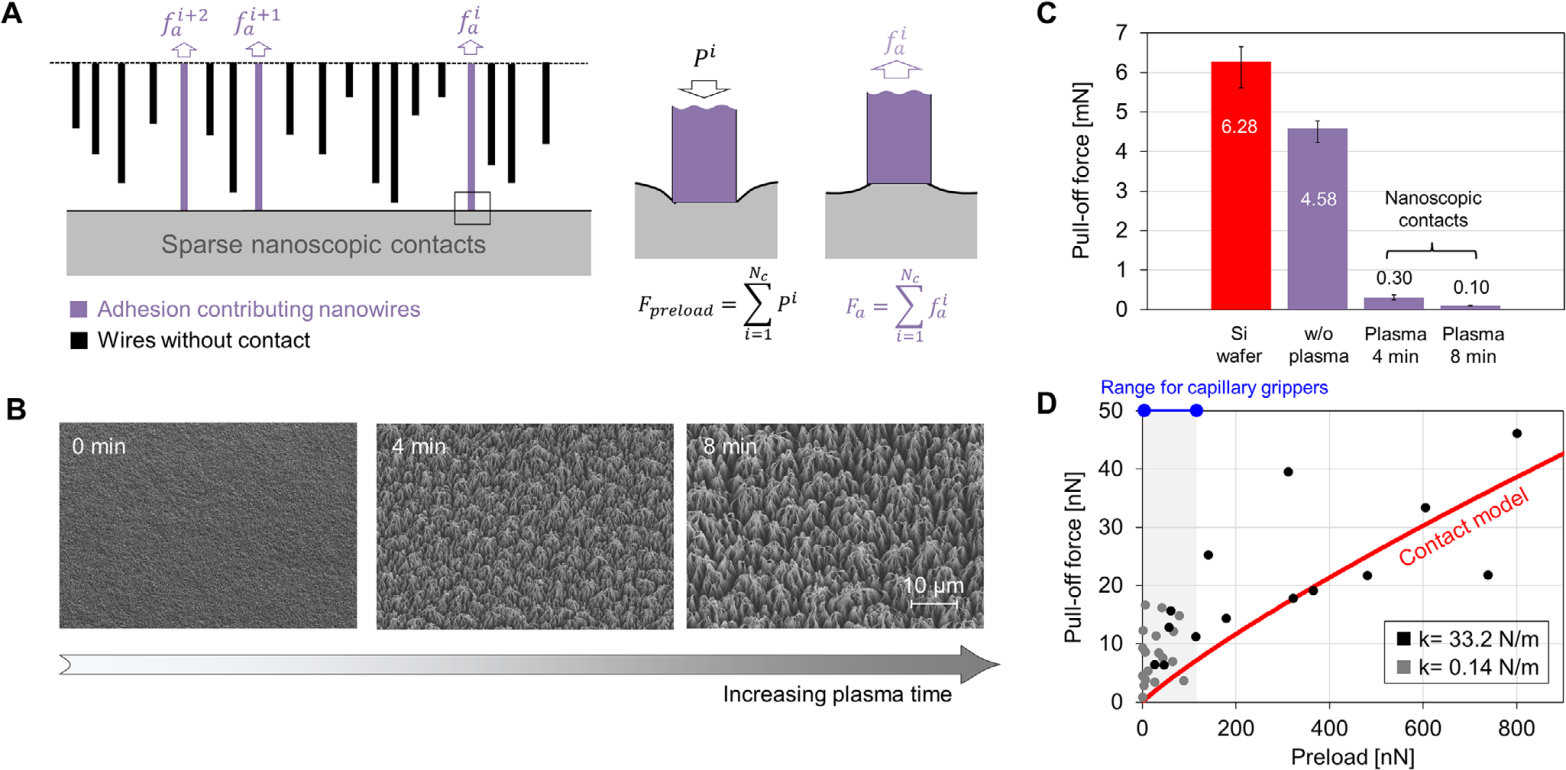
Contact mechanics behavior of the dry capillary gripper surface. A) Schematic illustrating the sparse and rough dry nanoporous gripper surface, which makes few nanoscale contacts with the counterpart surface. B) SEM images of gripper surfaces after plasma etching with different durations. Before plasma, the surface was composed of dense, entangled nanowires. After plasma etching, the entangled layer is removed, and the underlying vertically aligned nanowires appear on the top surface. As plasma etching continues, the surface becomes sparser and rougher. C) Measured pull‐off forces of bare Si wafer surface and dry nanoporous gripper surfaces after contact with PDMS. D) Predicted and measured pull‐off forces of dry nanoporous gripper surface according to different preload conditions. The dark and grey dots are the measured data using a rigid (k = 33.2 N m^−1^) and a compliant (k = 0.14 N m^−1^) AFM cantilever tip.

We propose that the mechanism behind the low adhesion of our gripper is the sparse distribution of nanocontacts. Our surface can be regarded as numerous randomly distributed asperities with sizes on the order of tens of nanometers. Since dry adhesion arises from intermolecular interactions acting over extremely short distances, only a small fraction of these asperities contributes to adhesion, **Figure** **5**A. The total adhesion force (*F_a_*) is the sum of attractive forces from individual small contacts (*f_a_*), and the key to achieving a small adhesion force is to minimize the number of contact asperities (*N_c_*).

We validated this hypothesis through pull‐off force measurements at both millimeter and micrometer scales, along with a nanocontact mechanical model. Three surfaces were created by varying plasma etching times, and we observed that surface porosity increased to ≈69% after 8 min of plasma exposure (Figure S23, Supporting Information). Compared to a reference adhesion measurement of 6.28 mN between Si and polydimethylsiloxane (PDMS), the plasma‐treated nanoporous surface exhibited a notably lower force of ≈0.1 mN against PDMS, Figure 5C. This substantial reduction cannot be attributed solely to changes in surface chemistry, as relatively high adhesion (4.58 mN) was measured with ZnO‐coated CNT samples that were not plasma‐etched (labeled as “w/o plasma” in the figure). Therefore, we confirmed that the introduction of sparse contacts significantly reduced adhesion.

Although a nanoporous surface is geometrically sparse and rough, resulting in low adhesion, the fibrillar asperities may deform when a high preload is applied, which increases the contact area and induces stronger surface adhesion by physical contact. Such adhesion dependency on preload can be found in any contact‐based transfer printing techniques, as the contact area between the solids increases with the preload.^[^ ^15^ ^]^ However, as discussed in the previous section, our nanoporous gripper can achieve high capillary adhesion without preload. The compressive force applied on the nanoporous surface is determined solely by the capillary force generated by the trapped liquid (Figure S24, Supporting Information). Therefore, only few rare contact points remain during the capillary pick‐and‐place, which will contribute to the extremely small adhesion force.

To examine the relationship between preload and adhesion in more detail, we performed single asperity adhesion tests using AFM. To measure the adhesion over a wide range, cantilevers with different spring constants (*k* = 33.2 N m^−1^ and 0.14 N m^−1^) were exploited. We found that pull‐off force of our nanoporous structure increases as greater preload is applied, Figure 5D. To explain this tendency, we used the contact mechanical framework (16, 35), where the height of a nanowire from the nominal plane (*l*) is assumed as a Gaussian probability density function (*ϕ*) as

(3) $$\phi \left(l\right)=\frac{1}{\sigma \sqrt{2\pi }}\;\exp\left(-\frac{l^{2}}{2\sigma^{2}}\right)$$

where *σ* is the standard deviation of the asperity heights. Accordingly, the probability of contact (*P_c_*) can be attained by integrating equation 3 as

(4) $$P_{c}=\frac{1}{2}\;\left\{1-\mathrm{erf}\left(\frac{d}{\sqrt{2}\sigma }\right)\right\}$$

where *d* is the distance between the nominal plane and the indenter surface. To simplify the situation, we assumed that the load‐displacement behavior of a single nanowire is linear, and the adhesion force produced by a nanowire is constant. Then, the expected values of preload (*F_preload_*) and pull‐off force (*F_pull‐off_*) with respect to *d* are expressed as

(5) $$F_{\mathrm{preload}}=\int 2\pi r\frac{k_{CNT}\sigma \varphi }{\sqrt{2\pi }}\left[\exp\left(-\frac{d^{2}}{2\sigma^{2}}\right)-\sqrt{\frac{\pi }{2}}\frac{d}{\sigma }\left\{1-\mathrm{erf}\frac{d}{\sqrt{2}\sigma }\right\}\right]dr\;$$

(6) $$F_{\mathrm{pull}-\mathrm{off}}=\int 2\pi r\varphi P_{CNT}\frac{1}{2}\left\{1-\mathrm{erf}\left(\frac{d}{\sqrt{2}\sigma }\right)\right\}dr\;$$

(7) $$d\;=D+R-\sqrt{R^{2}-r^{2}}$$

Here, *r* is the radial distance from the center of contact, *k_cnt_* is the stiffness of the nanowire‐solid contacts, *φ* is the number density of nanowires, *P_CNT_* is the adhesion force from a single nanowire‐solid contact, and *D* is the distance between the indenter tip and the nominal contact plane of the gripper surface (details in the supplementary material). Here, *σ*, *φ*, and *P_CNT_* were approximately estimated from SEM and AFM data, while *k_cnt_* was chosen to best explain the experimental results. Both experimental data and modeling results showed an increasing trend in pull‐off force as preload is increased. However, the analysis indicates that the predicted force applied to the nanoporous surface (116 nN in this case, Figure S24, Supporting Information) in capillary gripping can be controlled to remain in the region of extremely rare contact and low adhesion according to the contact model. Therefore, we can achieve the high adhesion of capillary force with minimum preload using the wet nanoporous surface and switch into extremely low adhesion of sparse nano‐contact after liquid evaporation.

### 2.5 Demonstrations of Ultragentle Manipulation

**Figure 6:**
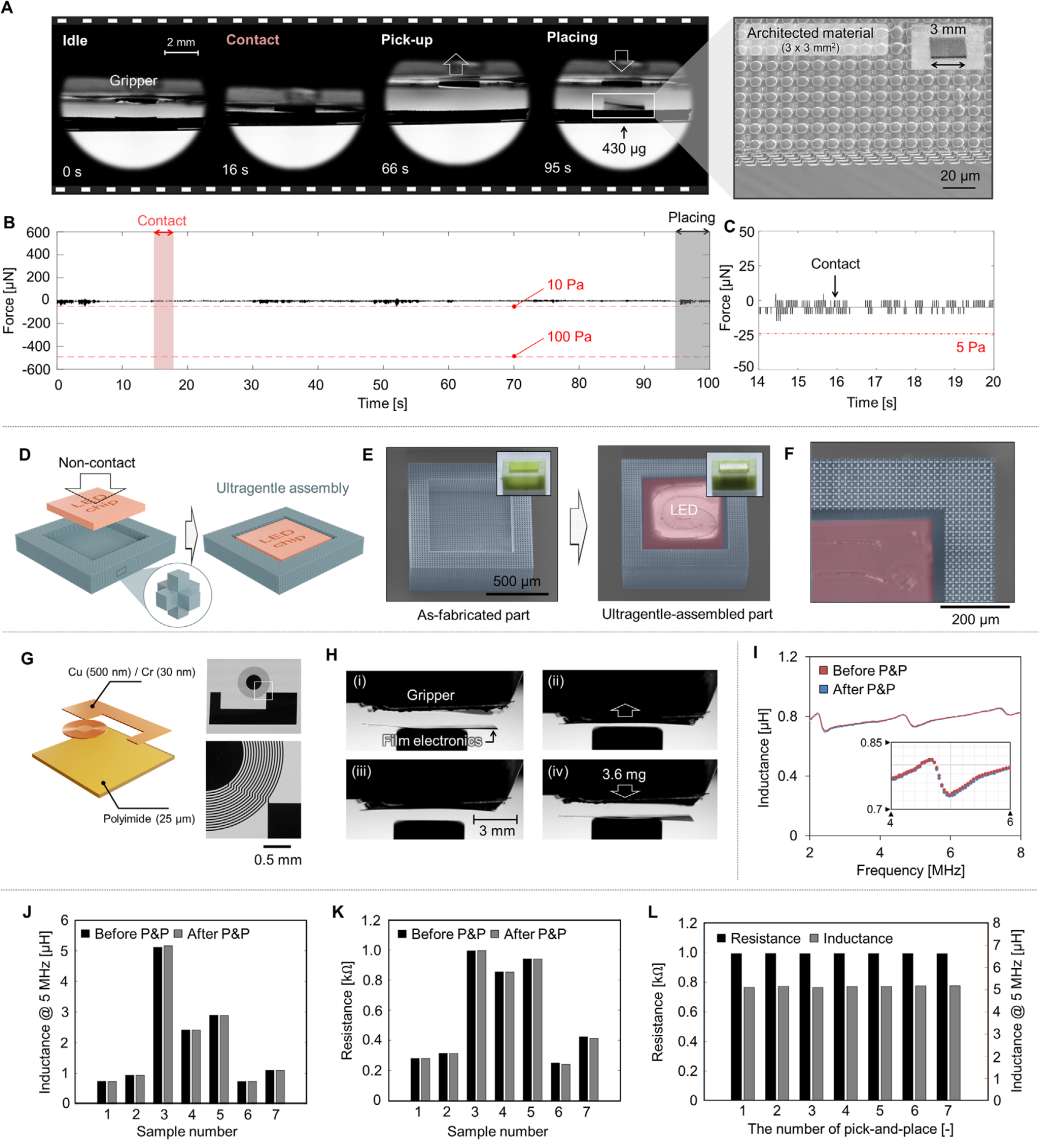
Demonstration of ultragentle transfer of delicate objects. A) Side‐view video snapshots of the pick‐and‐place (P&P) operation of a micro‐architected material, along with the corresponding SEM image. B,C) In situ force measurements during the P&P process. D) Schematic representation of an ultragentle microassembly process. E,F) SEM and optical images of the micro‐architected material before and after the assembly. G) Thin‐film electronic components utilized in the P&P demonstration. (H) Video snapshots of the P&P operation of the thin‐film electronics. I–L) Measured inductance and resistance of the thin‐film electronics before and after the P&P process.

Ultragentle manipulation can be utilized to transport structurally fragile materials without causing damage. As a first example, we demonstrate the pick‐and‐place of a micro‐architected material (µAM, 3×3 mm^2^) using our gripper (**Figure** **6**A and Movie S6, Supporting Information). Although µAMs offer a wide range of applications with fascinating mechanical and optical properties,^[^ ^50^ ^]^ their integration into functional devices has seldom been explored. Throughout manipulation of the µAM, we monitored the contact force in real‐time using a cantilever force sensor, observing that the maximum nominal pressure remained below 5 Pa (Figure 6B,C). Additionally, we manipulated a small LED chip (600×600 µm^2^) into a µAM structure (Figure 6D,E). As depicted in Movie S7 (Supporting Information), the placement process was entirely contact‐free with the receiver substrate, avoiding structural damage (Figure 6F).

Thin electronics pose challenges for pick‐and‐place processes and heterogeneous integration due to their vulnerability to performance degradation from bending. We demonstrated our gripper's capabilities toward this challenge by transporting a thin inductor (25 µm thick) (Figure 6G,H, Table S1, Supporting Information). We evaluated the device's performance before and after transfer through impedance measurements (Figure 6I–L). After the pick‐and‐place process, no significant inductance differences were noted within the 2 to 8 MHz frequency range (Figure 6I), with an average inductance variation below 0.5%. When comparing average inductance and resistance values across seven samples, minimal differences of less than 0.05 µH in inductance and less than 10 Ω in resistance were observed (Figure 6J,K). After seven pick‐and‐place cycles, only ≈1.5% change in inductance and ≈0.02% change in resistance were recorded (Figure 6L).

During the solid‐contactless release process, placement errors may occur as the object falls through the air and becomes influenced by other forces (e.g., electrostatic forces). This can be greatly mitigated by minimizing the distance between the receiving surface and the gripper (Figures S25–S27, and Movie S8, Supporting Information), or by reducing the size of the gripper (Figure S28, Supporting Information). This strategy can also be extended to simultaneous pick‐and‐place of multiple objects. We fabricated a capillary gripper with a cylinder array pattern with a diameter of 1.5 mm and a spacing of 1.5 mm. Using this, we demonstrated that three separated small capacitor chips (0.5 mm x 1 mm in size) can be picked‐and‐placed simultaneously with a placement error of less than 0.16 mm (Figure S29 and Movie S9, Supporting Information).

Our capillary gripper requires minimal contact pressure and is insensitive to surface contamination, which enables long‐term operation. We demonstrated that 45 repeated pick‐and‐place cycles were performed with 100% success rate, and that even 0.6 mg aluminum foil and 1.4 mg small capacitor chips could be released without solid contact after these cycles (Figures S30 and S31 and Movie S10, Supporting Information).

In conclusion, we developed a novel capillary gripper design featuring significantly improved adhesion tunability compared to mechanical contact‐based approaches, along with negligible preload sensitivity. Comprehensive multiscale adhesion testing and modeling validated the gripper performance and identified critical operational parameters. We believe our work opens new possibilities for the delicate manipulation of microscopic materials across diverse applications. For instance, precise manipulation using a nanoporous capillary gripper can enable low‐damage handling and integration of thin‐film electronics, increasingly important in advanced flexible electronics.^[^ ^6^ ^]^ Furthermore, given its noncontact placement capability at millimeter scales, our gripper may facilitate the integration of architected materials into various functional devices. Future research exploring additional attractive forces, such as electrostatic and magnetic forces, and active control of capillary force using electrowetting could further enhance the scope and effectiveness of ultragentle pick‐and‐place manipulation. For example, the adhesion can be significantly enhanced by appropriately charging the capillary gripper, thereby simultaneously leveraging electrostatic interactions. Electrostatic force is a force proportional to the area and when combined with capillary force, it can provide an adhesion force several times stronger on the sub‐millimeter scale.

## 3 Experimental Section

### Fabrication of the Capillary Gripper

We deposited a catalyst layer composed of 10 nm of Al_2_O_3_ and 1 nm of Fe on a Si wafer by e‐beam evaporation (Alpha‐plus, Korea) for CVD of a VACNT forest. The catalyst‐coated wafer was then placed in a tube furnace, where the hydrocarbon source (C_2_H_4_), hydrogen, and helium were introduced according to the recipe (Table S2, Supporting Information). We maintained a temperature of 775 °C during the growth process. Subsequently, we performed laser drilling using a femtosecond laser (SM‐LMM‐9100, SM Tech, Republic of Korea) to create liquid channels on a 65 µm‐thick polyimide tape (SOYU T&E Corp, Korea). The as‐grown VACNT forest was then transferred to the adhesive side of the polyimide tape. To ease the delamination of CNTs from the wafer substrate, we heated the VACNT‐grown wafer at 490 °C under an oxygen flow of 30 mL min^−1^ for 20 min. During this step, oxygen has been reported to weaken the bonds at the catalyst‐CNT interface, which in turn reduces the interfacial adhesion.^[^ ^51^ ^]^ To reduce the surface porosity, we conducted plasma etching on the CNT surface at a power of 80 W for 4 min (Ultech, Korea). The process was carried out under SF_6_ and Ar flow rates of 12 and 4 sccm, respectively. Finally, a ZnO film was deposited through 200 cycles of ALD. Zn(C_2_H_5_)_2_ and H_2_O were used as the precursor and oxidant, respectively. The chamber temperature was maintained at 155 °C throughout the deposition.

### Adhesion Measurement

Depending on the magnitude of the applied force, we used either an AFM or a custom‐built adhesion tester for pull‐off force measurements. For microscopic adhesion testing, we employed a benchtop AFM (flexAFM, Nanosurf) and cantilevers with colloidal probes that had a force constant of 0.1–0.2 N m^−1^ (CP‐qp‐CONT‐SiO2, sQube, USA) or 30–40 N m^−1^ (CP‐NCH‐SiO, sQube, USA). The force constants were calibrated using a vibrational method, as described in the literature.^[^ ^52^ ^]^ The diameter of the colloidal probes used in all measurements was 6.62 µm.

In contrast, a custom‐built cantilever‐type adhesion tester was used for millimeter‐scale pull‐off measurements. We used a precision noncontact displacement sensor (ILD1420‐10, Micro‐Eplison), operating at a frequency of 2 kHz and with a repeatability of 0.5 µm. A 0.1 mm‐thick nickel plate was used as the cantilever. To precisely control the retraction displacement, we used a high‐resolution 2‐axis stage (Tribolab, Bruker). The retraction speed was set to 0.05 mm s^−1^ for Figure 4C–E and 0.1 mm s^−1^ for Figure 5C. For the millimeter‐scale pull‐off test shown in Figure 5C, we used a 3 mm‐diameter flat cylinder coated with a thin PDMS layer. A ball bearing was added to improve alignment (Figure S32, Supporting Information). We prepared PDMS by mixing the base and curing agent at a 10:1 ratio and curing it at 80 °C for 3 h.

### Fabrication of Objects for Ultragentle Transfer

To demonstrate the capability of ultragentle transfer, we fabricated micro‐architected materials and thin‐film electronics. Micro‐architected materials were produced using a high‐resolution 3D printer (nanoOne, UpNano) via two‐photon polymerization with a commercial resin (UpBrix, UpNano). A laser power of 20 mW was maintained throughout the printing process. During the development, we immersed the samples in propylene glycol monomethyl ether acetate for 2 h and rinsed them with isopropyl alcohol.

Thin‐film electronics were fabricated by conventional photolithography on thin polyimide films (Alphaflon, Korea). First, a commercial photoresist (AZ5214, MicroChemicals, Germany) was spin‐coated at 2000 rpm and then baked at 110 °C. A precisely manufactured photomask (Corelink, Korea) was then aligned and exposed to 150 mJ cm^−2^ with a precision aligner and UV lamp (Midas Systems, Korea). The exposed photoresist layer was developed using AZ 300MIF solution. Subsequently, 500 nm of Cu and 30 nm of Cr were deposited on the patterned photoresist layer via e‐beam evaporation (Alpha‐plus, Korea), and the residual photoresist was removed in ST‐1023 stripping solution (Jeonyoung, Korea). The electrical characteristics of the fabricated electronics were evaluated using an LCR meter (IM3536, HIOKI).

## Conflict of Interest

The authors declare no conflict of interest.

## Supporting information



Supporting Information

Supplemental Movie 1

Supplemental Movie 2

Supplemental Movie 3

Supplemental Movie 4

Supplemental Movie 5

Supplemental Movie 6

Supplemental Movie 7

Supplemental Movie 8

Supplemental Movie 9

Supplemental Movie 10

## Data Availability

The data that support the findings of this study are available from the corresponding author upon reasonable request.
