# Supplementary material for: Nanoporous Capillary Gripper for Ultragentle Micro‐Object Manipulation
Source: Adv Sci (Weinh). 2025 Jun 25;12(36):e08338. doi: 10.1002/advs.202508338 (PMC12463134; doi:10.1002/advs.202508338)
Supplement: Supplementary file 1 — Supporting Information [file ADVS-12-e08338-s002.docx]

Supporting Information

Nanoporous capillary gripper for ultragentle micro-object manipulation

Seong Jae Kim, Taehoon Kim, Hyun Jun Ryu, Ji-hun Jeong, A. John Hart, Sanha Kim*

**
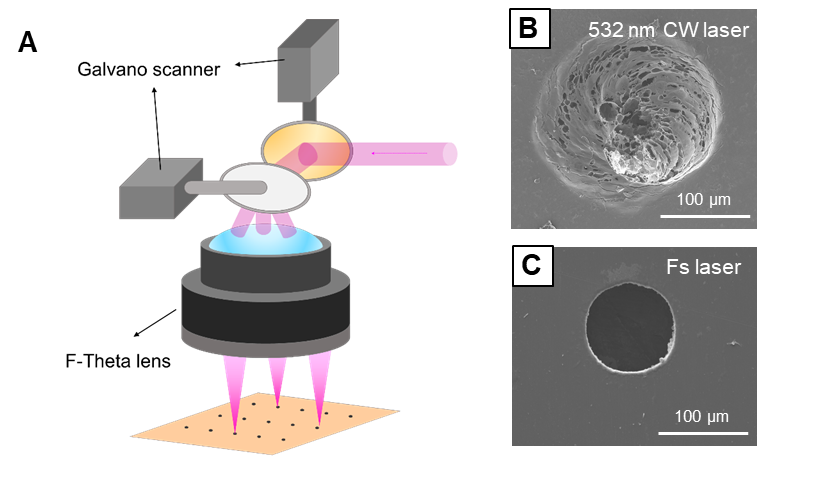
**

**Figure S1.** Schematic illustration of a femtosecond laser drilling process. (A) Configuration of the equipment used in the process, laser drilling results from (B) a conventional CW laser, and (C) a femtosecond pulse laser

**
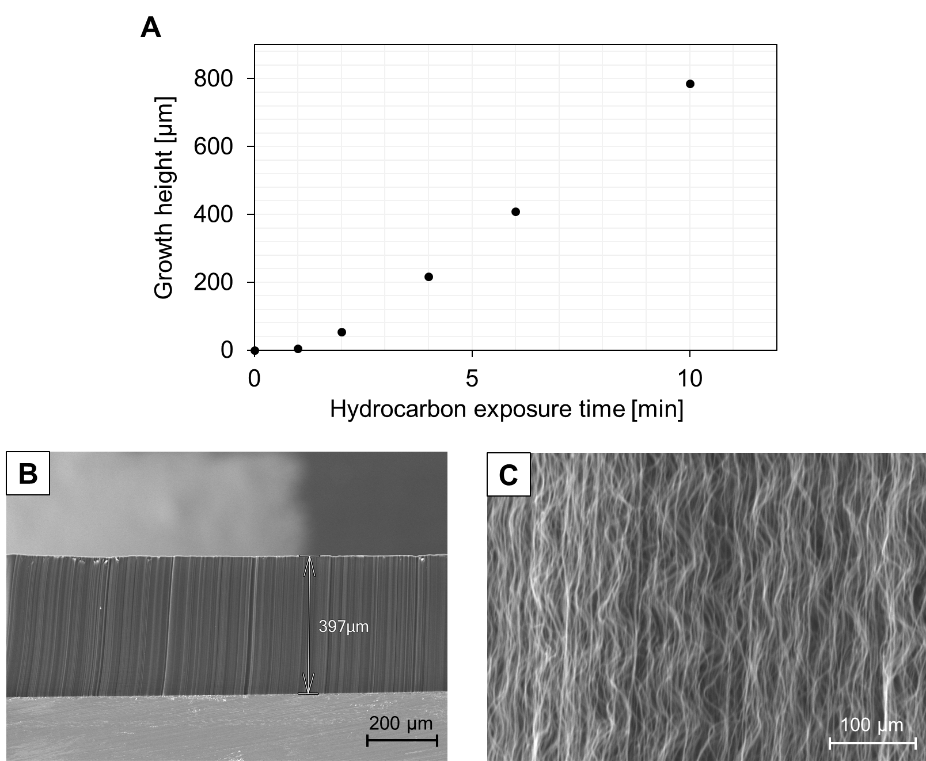
**

**Figure S2.** As-grown vertically-aligned carbon nanotube forest. (A) Growth height to the hydrocarbon exposure time, (B)-(C) SEM images of the as-grown vertically-aligned carbon nanotube (VACNT) forest with 5 minutes of hydrocarbon exposure time.

**
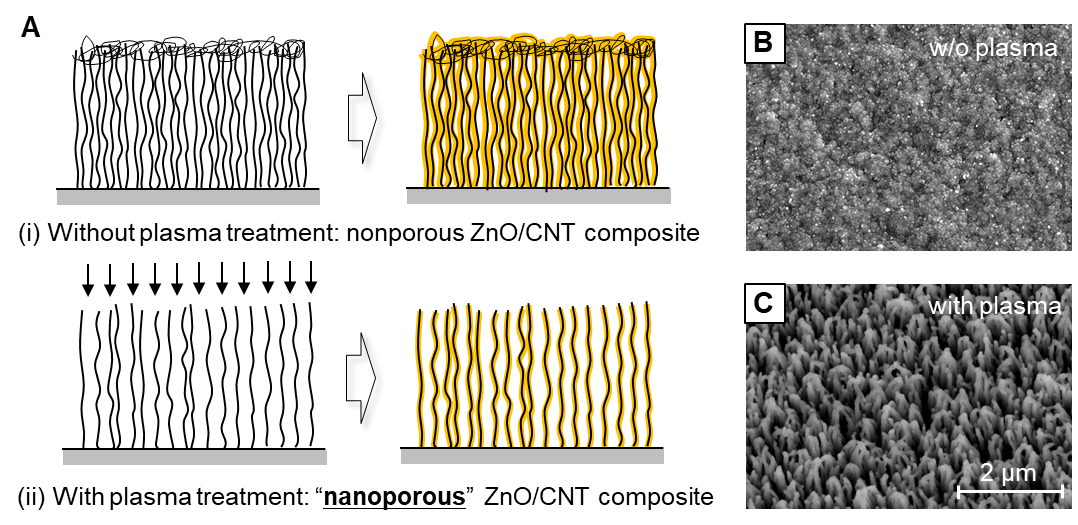
**

**Figure S3.** Effect of plasma treatment for an optimal surface porosity of the gripper. (A) Schematic illustration explaining the effect of plasma treatment, SEM images of ZnO-coated VACNTs (B) without plasma etching, and (C) with 4 minutes of plasma etching.


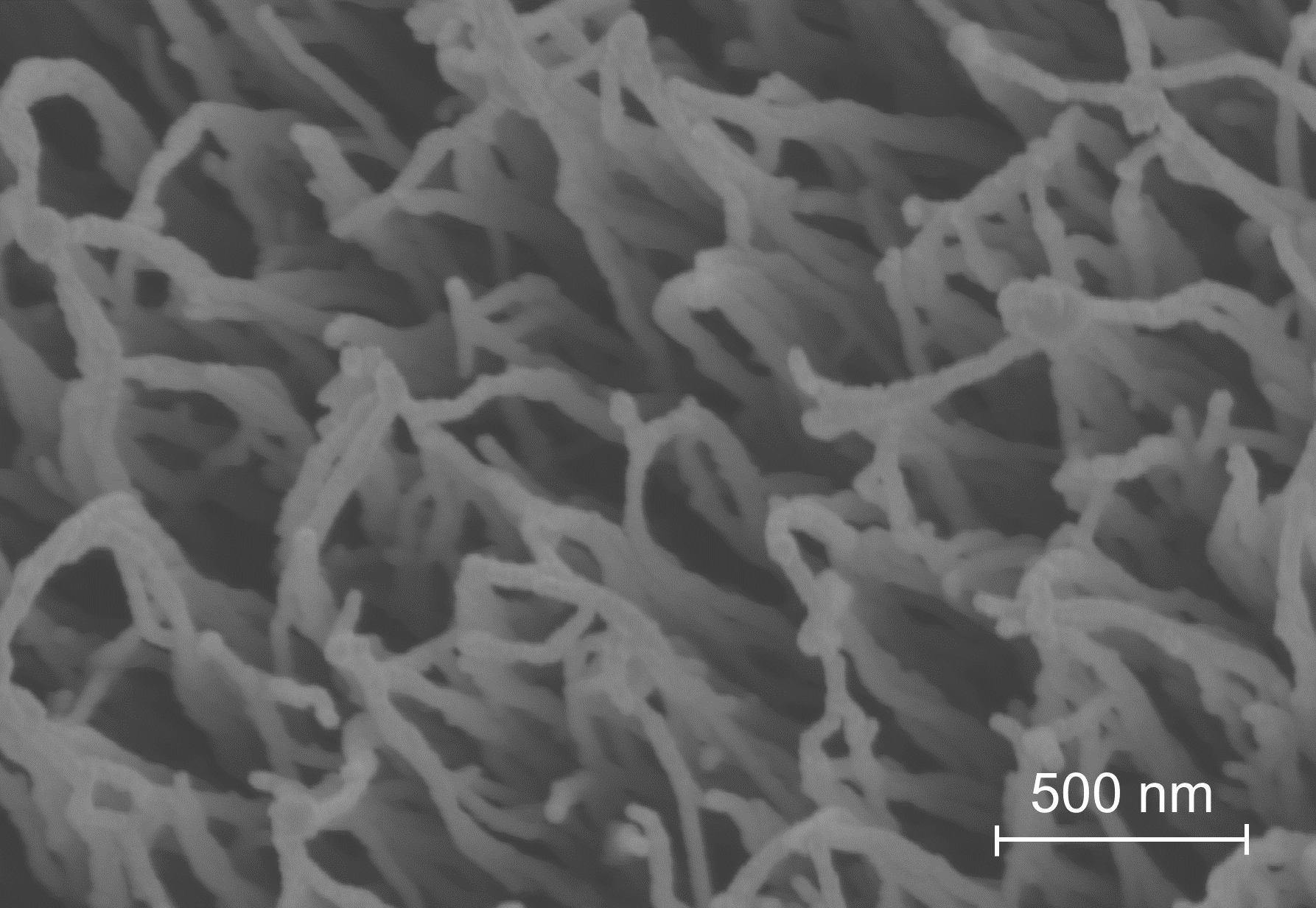


**Figure S4**. Close-up SEM image of a ZnO-coated VACNT surface.


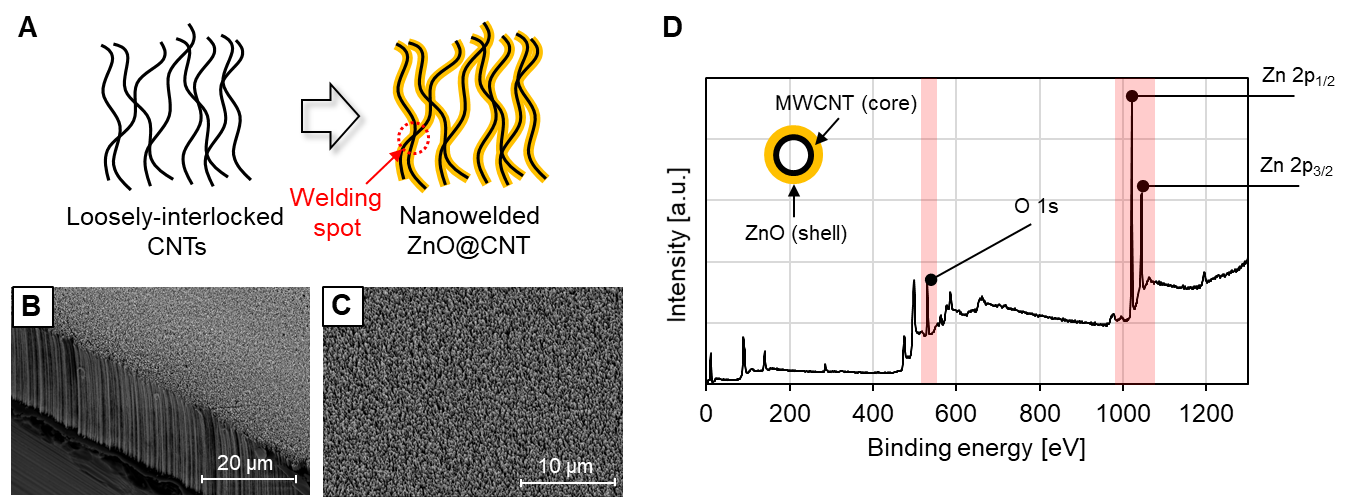


**Figure S5**. Effect of atomic layer deposition (ALD) of ZnO for the improvement of mechanical robustness. (A) Schematic illustration showing the structural change after ALD, (B)-(C) SEM images of the ZnO-deposited VACNT forest after ethanol evaporation, (D) X-ray photoelectron spectroscopy result depicting the successful ZnO deposition on a VACNT


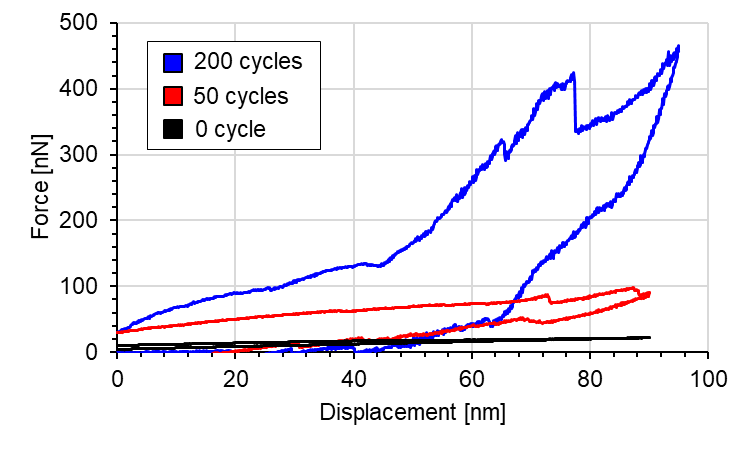


**Figure S6**. Representative nanoindentation data obtained from gripper surfaces
treated with 0, 50, and 200 cycles of ZnO atomic layer deposition


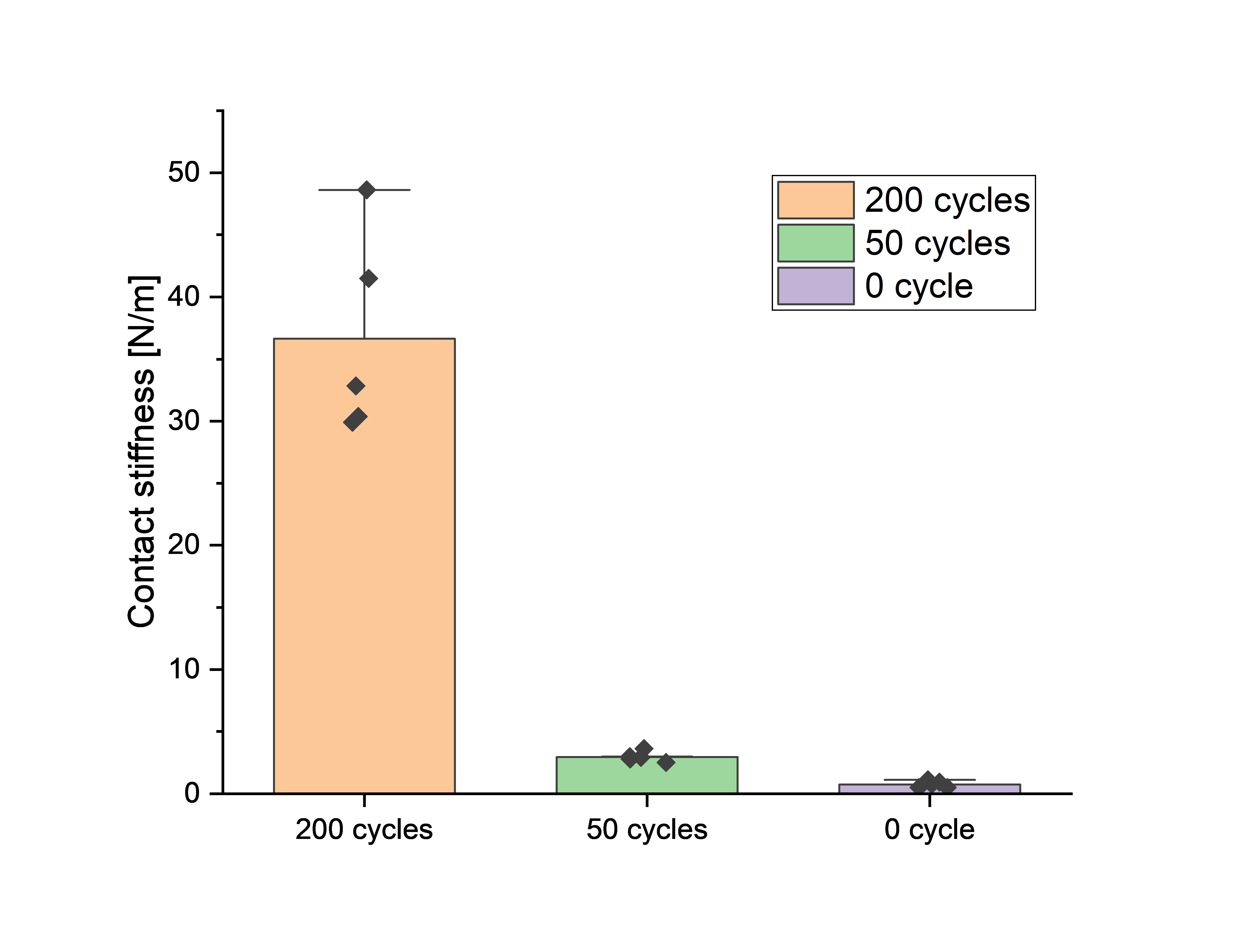


**Figure S7**. Contact stiffness of the gripper surfaces
after 0, 50, and 200 cycles of ZnO-ALD

Atomic force microscopy (AFM) was used to perform nano/microscale indentation tests. A cantilever equipped with a 6.62 μm SiO_2_ hard microsphere was employed. In all tests, the displacement was controlled to 90 nm. A contact force of 30 nN was applied for samples with 200 and 50 ALD cycles, and 10 nN was used for the 0-cycle sample. Contact stiffness was calculated from the initial slope of the retracting curve. All measurements were repeated five times.

The nanoindentation results showed that the contact stiffness significantly increased upon introduction of ZnO-ALD. A contact stiffness of 36.6 N m^-1^ was observed after 200 ALD cycles, 3.0 N m^-1^ after 50 cycles, and 0.75 N m^-1^ without coating. The contact stiffness increased by more than 48-fold after coating, providing clear evidence of mechanical enhancement.


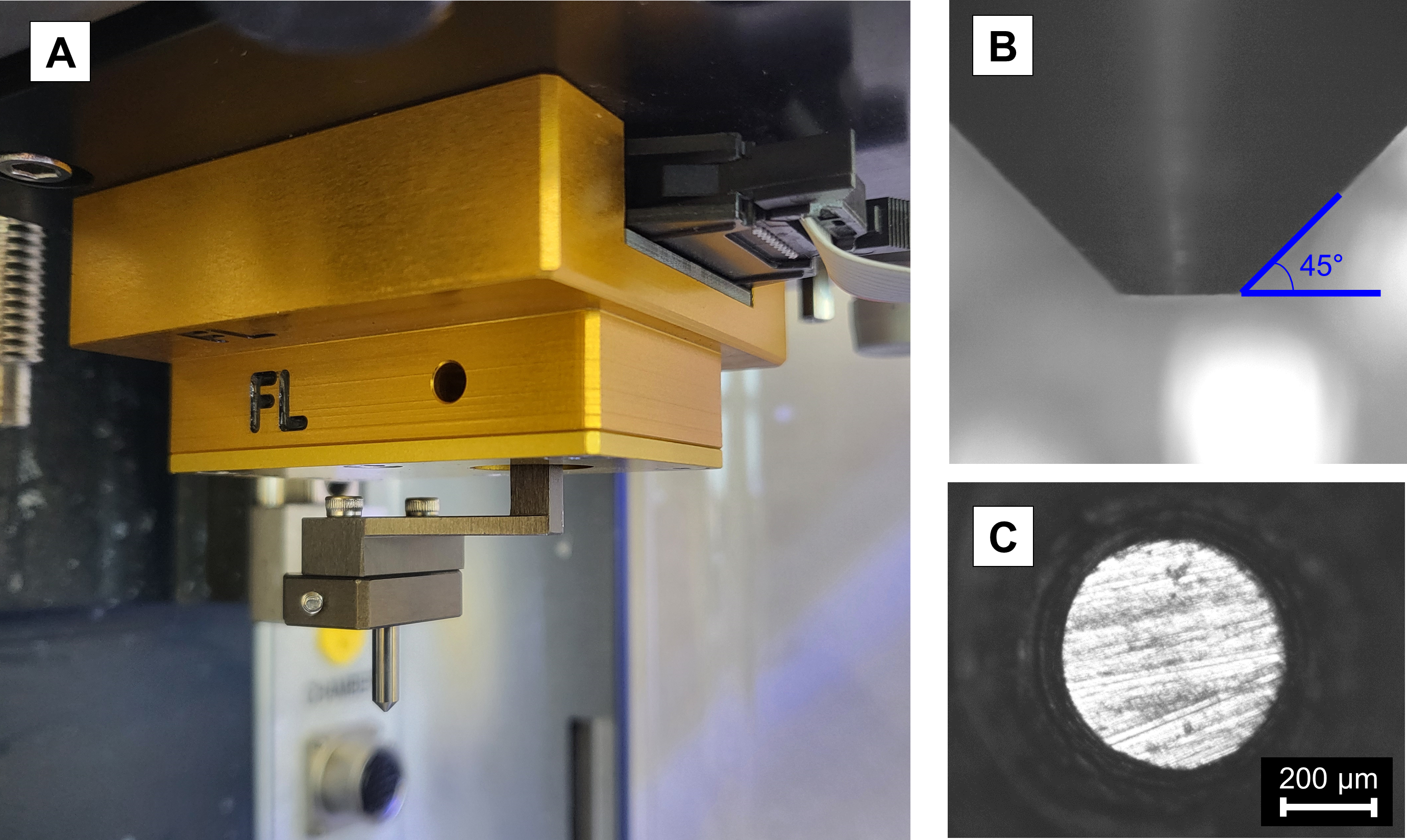


**Figure S8**. Photograph of the truncated cone indenter and the load cell
used in the mechanical testing setup.


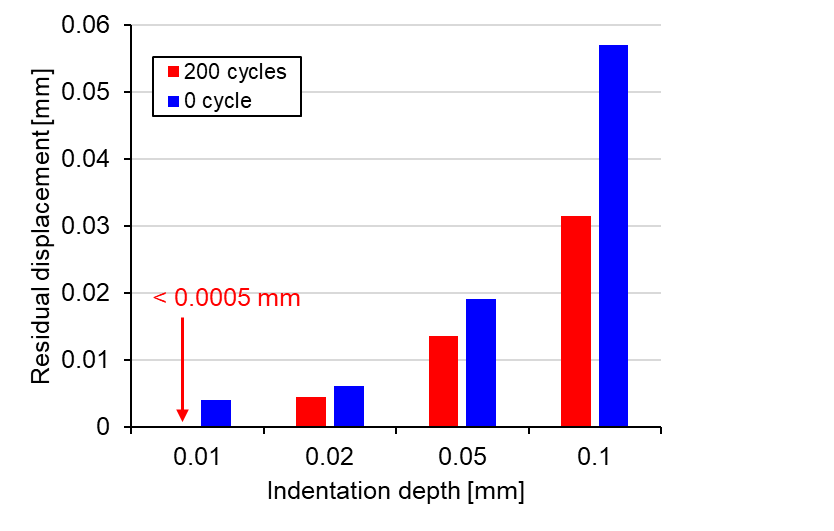


**Figure S9**. Residual displacements at various indentation depths
with and without 200 cycles of ZnO-ALD.

One important characteristic of mechanical robustness is to show that the residual displacement (permanent deformation) after indentation is small. Experimental results show that the introduction of ALD coating contributes to reducing residual displacement and improving mechanical robustness. We measured residual displacement at four different indentation depths (0.01 mm, 0.02 mm, 0.05 mm, and 0.1 mm). Through experiments, we confirmed that it exhibits a very small residual displacement close to 0 with indentation depth of 10 μm. As the indentation depth increases, the residual displacement tends to increase. This may be because irreversible damage may occur inside the nanoporous membrane. However, when comparing before and after ALD, the residual displacement always became smaller after ALD. These experimental results support that the introduction of ZnO-ALD also enhances the mechanical robustness at the macroscale.


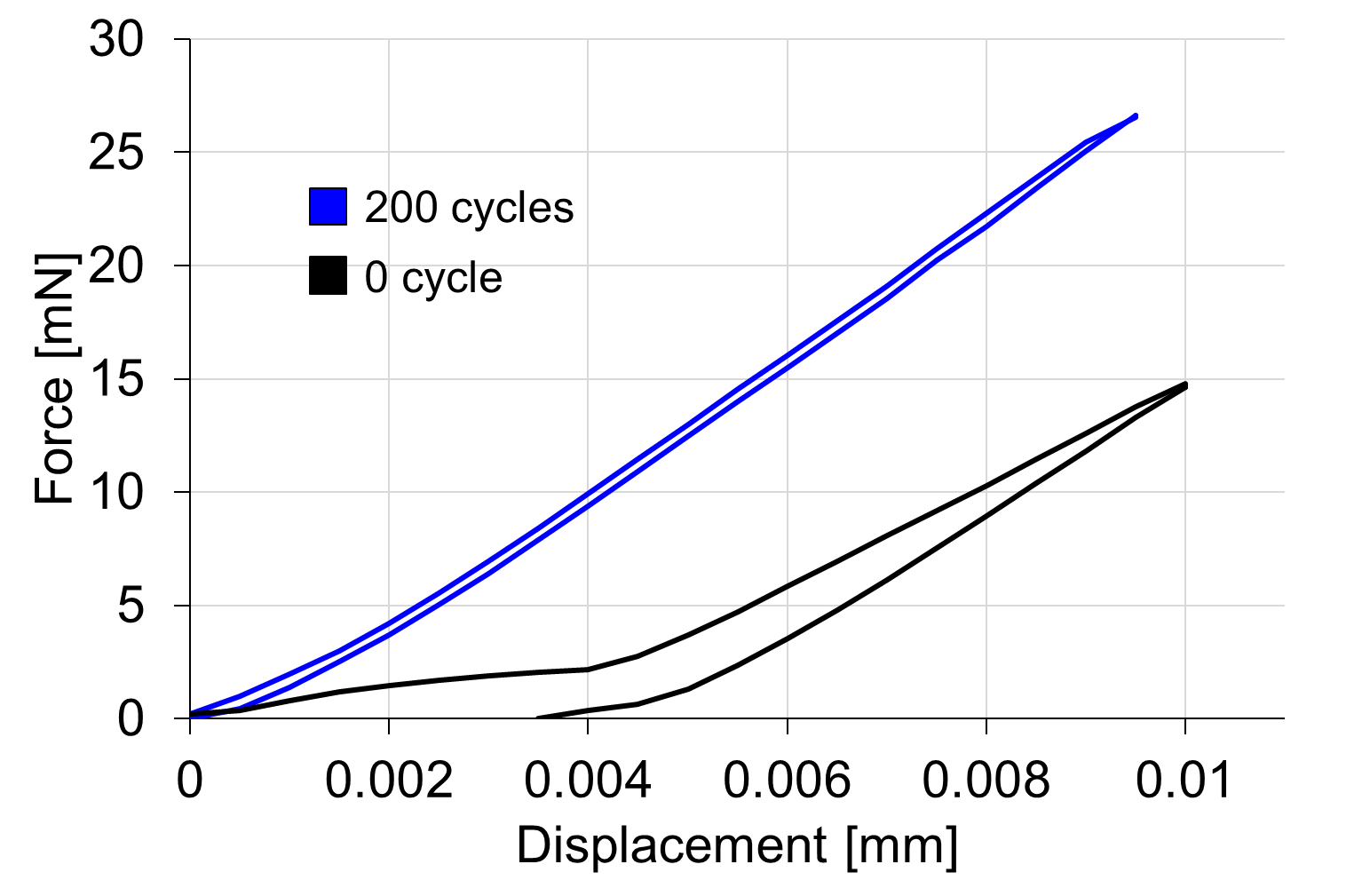


**Figure S10**. Macroscopic indentation behavior of grippr surfaces
 with and without 200 cycles of ZnO-ALD.


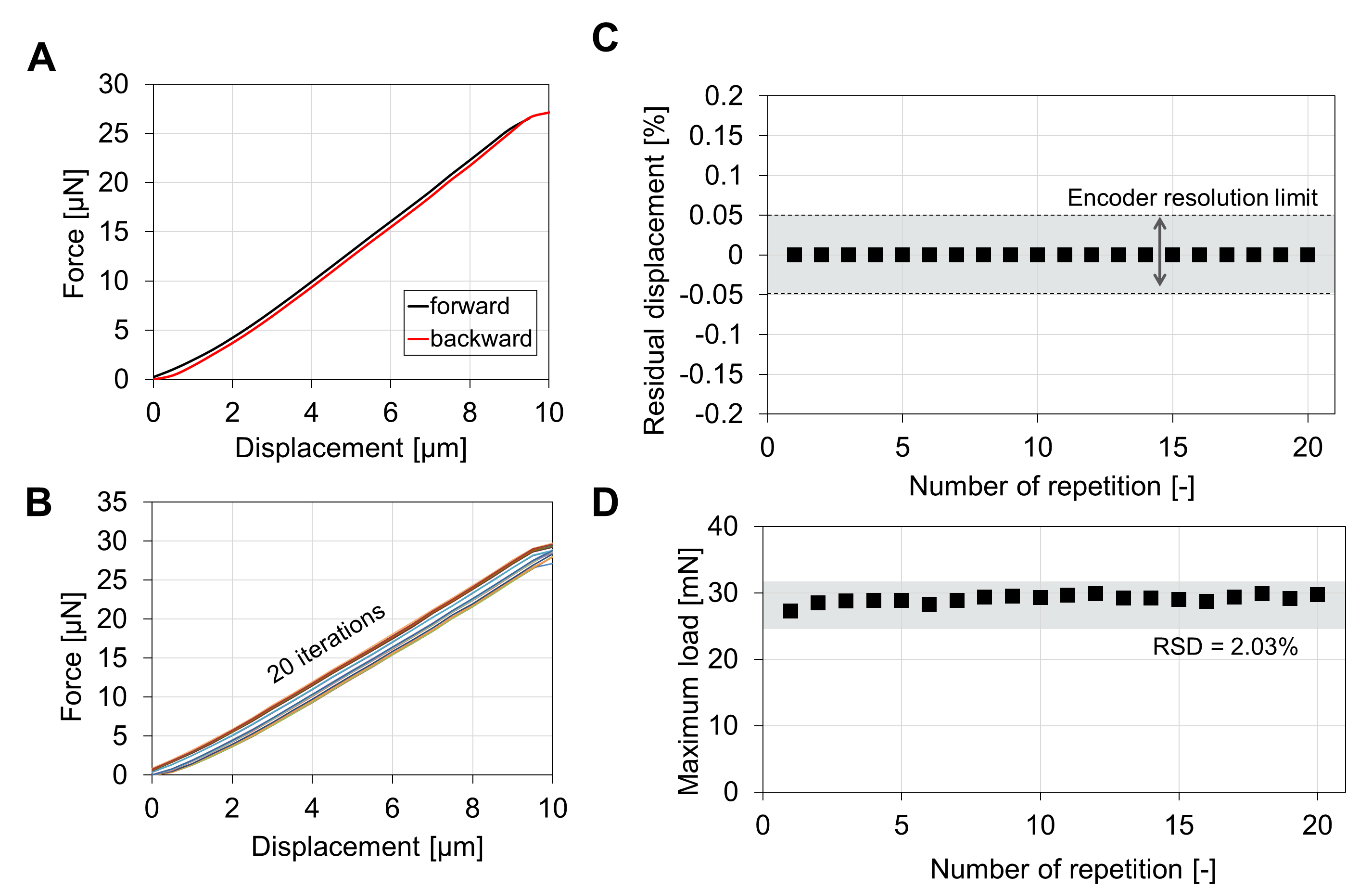


**Figure S11**. Macroscopic indentation tests showing negligible residual displacement. (A) Load–displacement curve from a single indentation; (B) load–displacement curve from 20 iterative indentations; (C) residual displacement and (D) maximum load as a function of the number of iterative indentations.

We confirmed that a small indentation of 0.01 mm exhibited negligible residual displacement even after 20 repetitions, which was smaller than the resolution of the encoder in the automated stage. This indicates that an indentation depth of 0.01 mm falls within the nearly perfectly elastic regime for ZnO-coated VACNT. This property is highly advantageous for practical applications, as capillary grippers must withstand repeated use.


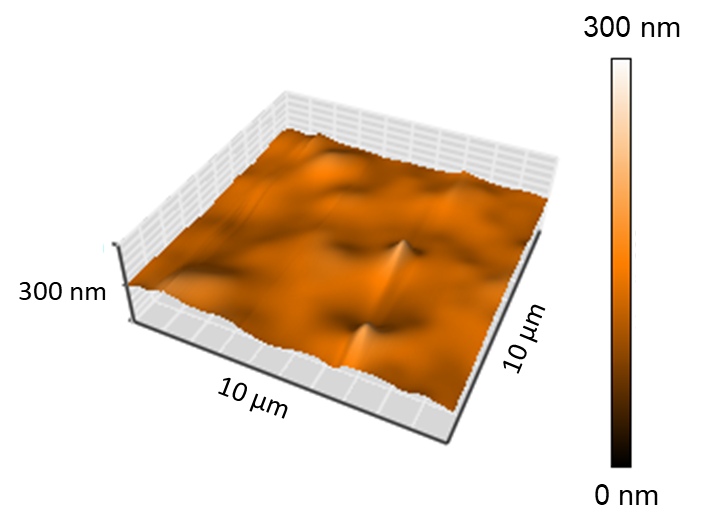


**Figure S12**. AFM surface scanning results of the tetradecane-wetted gripper surface
with a surface roughness of 6.1 nm

**
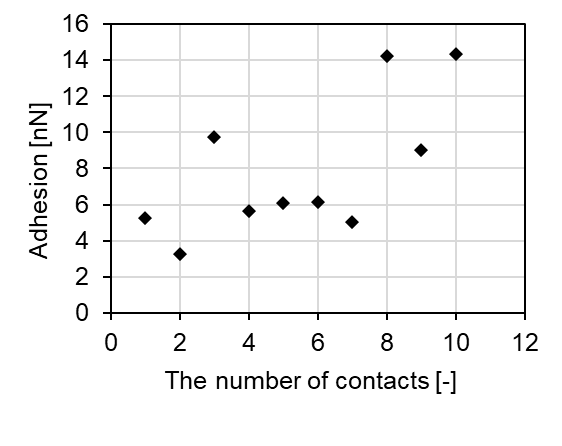
**

**Figure S13.** Repeated AFM adhesion (pull-off force) measurements from a dry gripper surface.


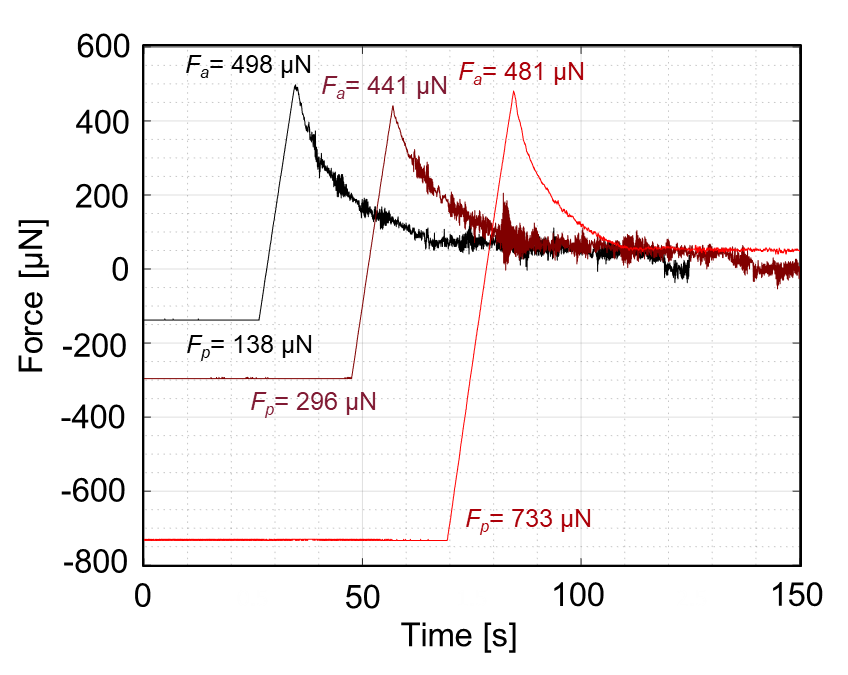


**Figure S14.** Pull-off force (*F_a_*) measurement with different preload forces (*F_p_*)
with a contact diameter of 1.5 mm.


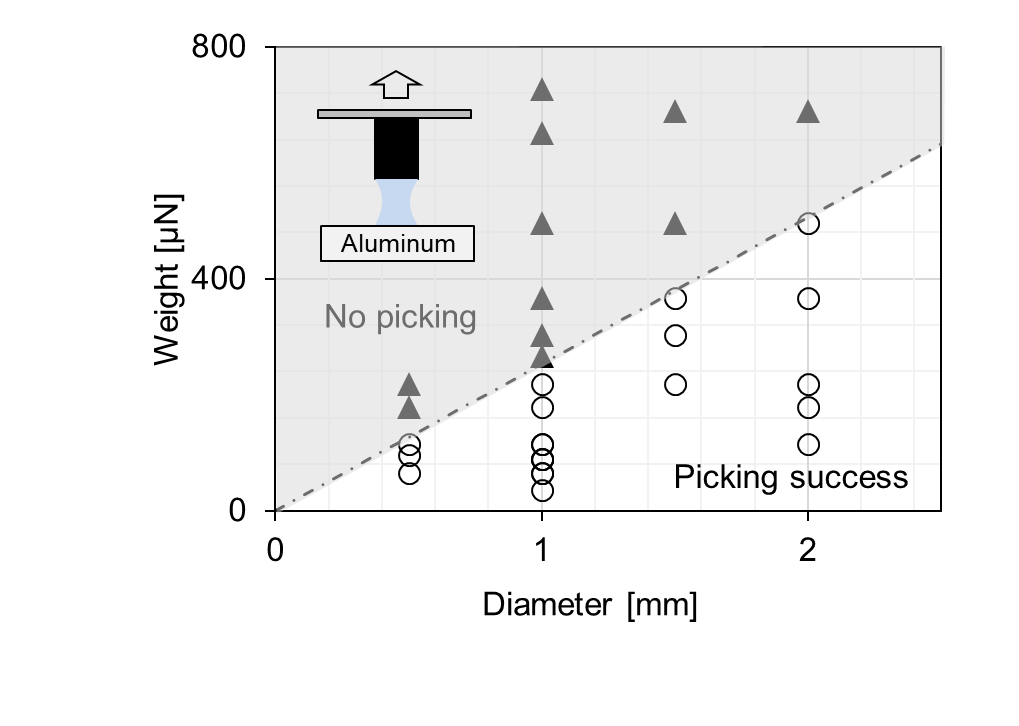


**Figure S15.** Pick and place map of a capillary gripper with a large volume of liquid bridge.


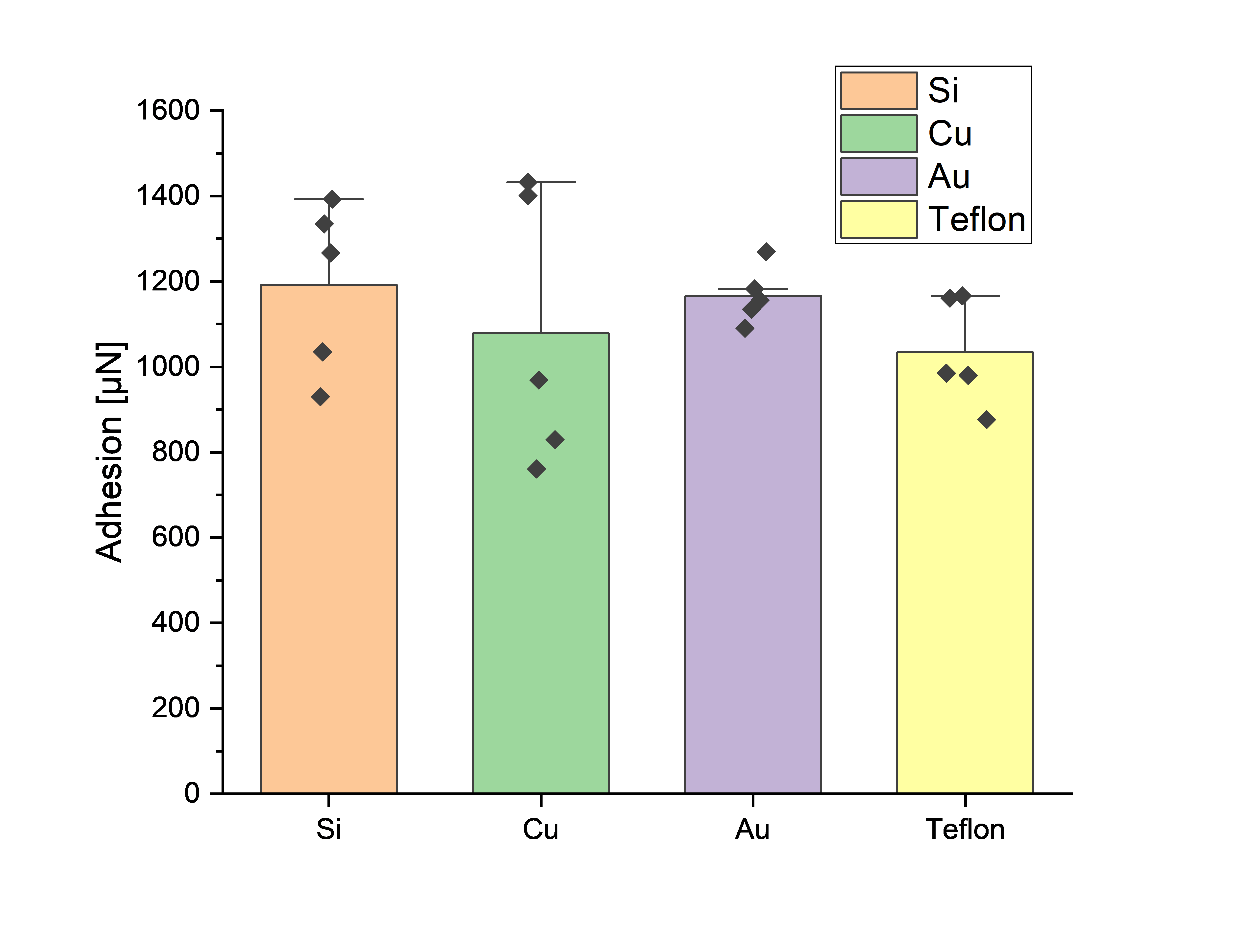


**Figure S16**. Comparison of capillary adhesion on different contacting surfaces

Si wafer; Si wafer with 1 µm Cu deposition (labeled 'Cu' in the plot); Si wafer with 100 nm Au deposition (labeled 'Au' in the plot); and Teflon (Nitto, Japan) were used as contacting surfaces. To enable capillary adhesion, 10 µL of ethanol was applied to the surface, and the system was allowed to idle for 80 seconds before measuring the adhesion force.


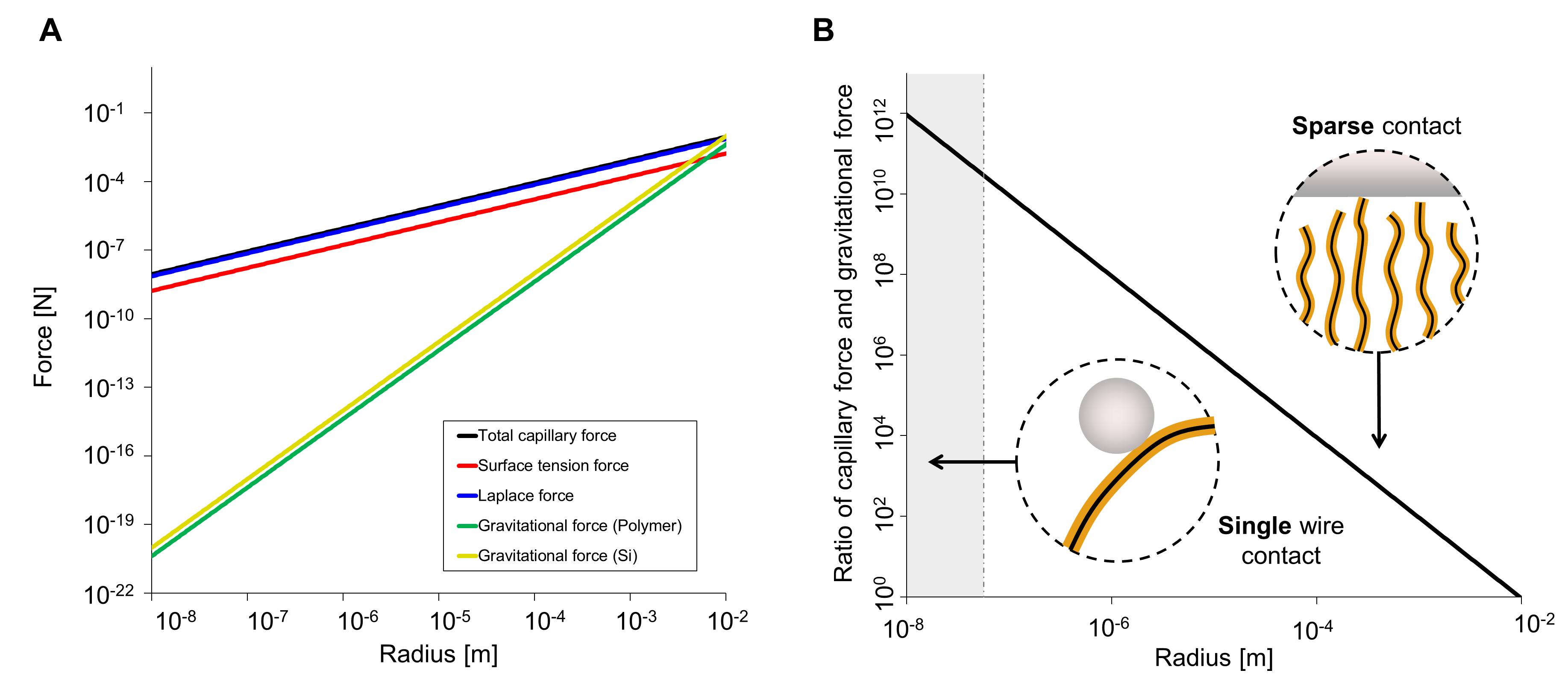


**Figure S17**. Comparison of capillary force and gravitational force as a function of length scale. (A) Capillary force and gravitational force at different length scales. (B) Ratio of capillary force to gravitational force at different length scales.


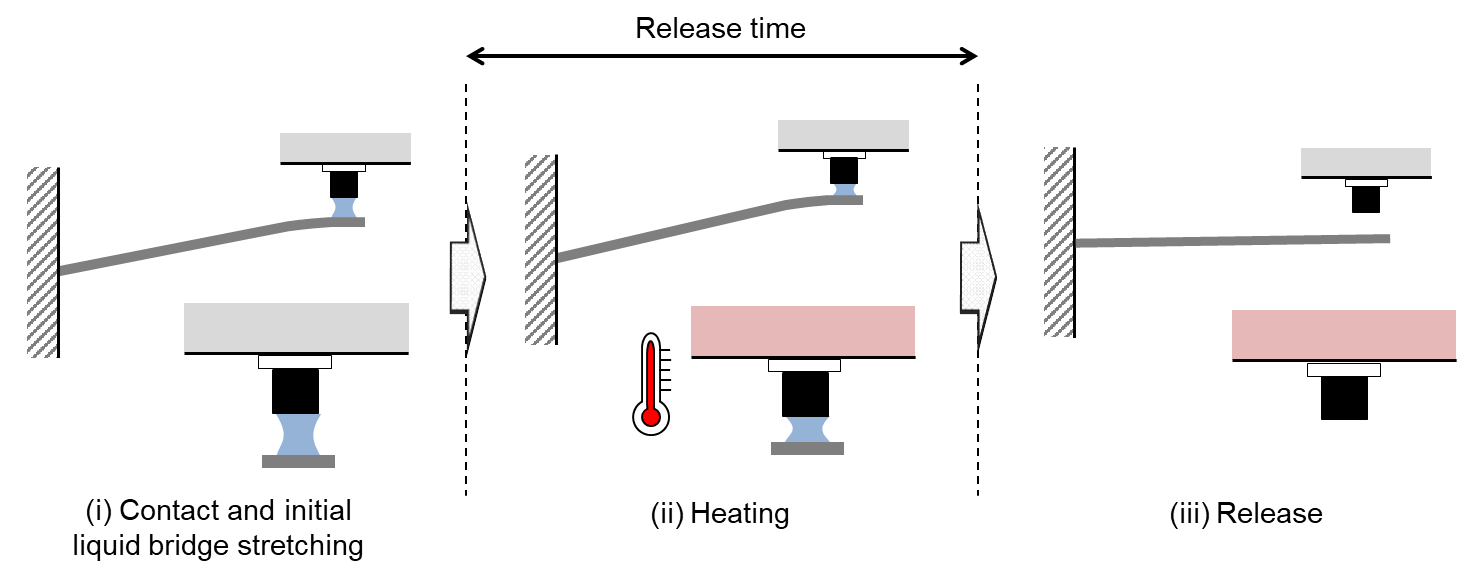


**Figure S18.** Configuration of the experiment for a release time measurement.

The diameter of the gripper, relative humidity, and room temperature were 2.5 mm, 50%, and 25 ˚C, respectively.


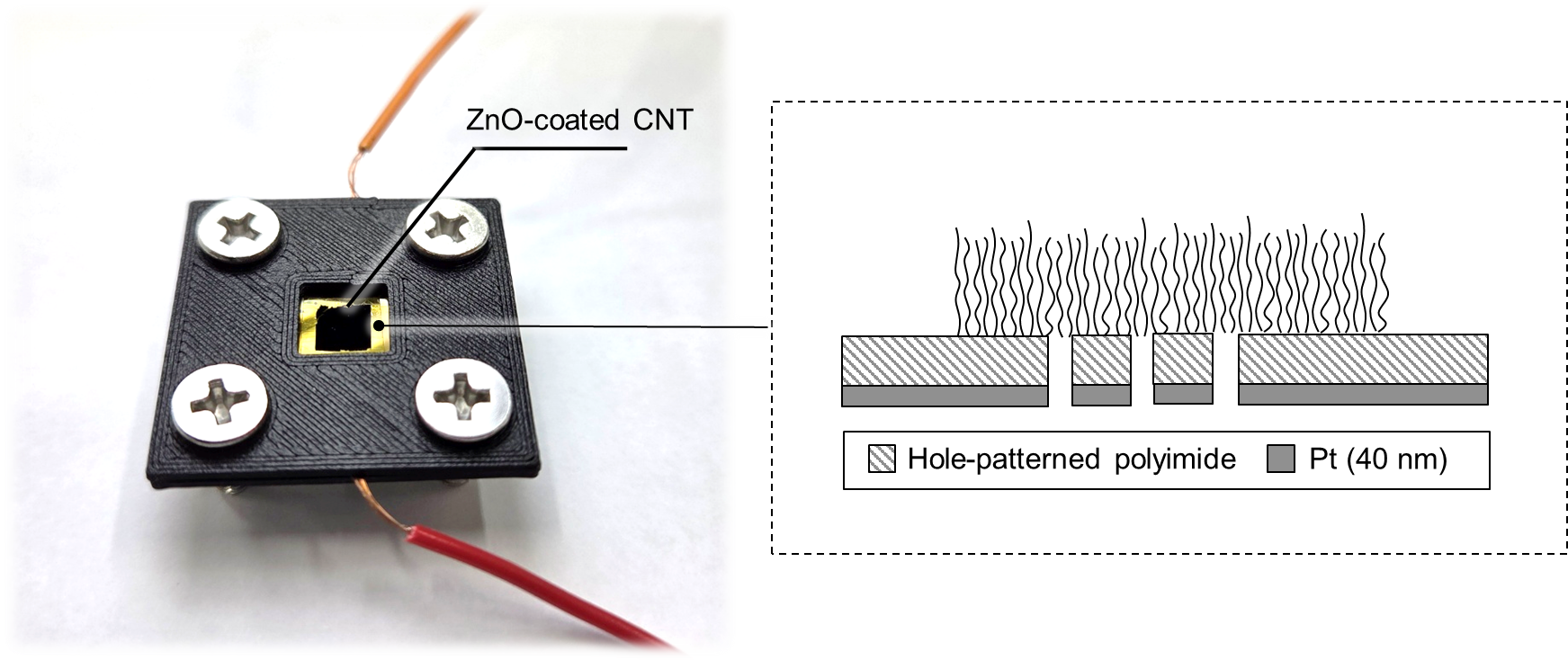


**Figure S19**. Photograph of a capillary gripper with a Pt-coated polyimide backing enabling Joule heating.


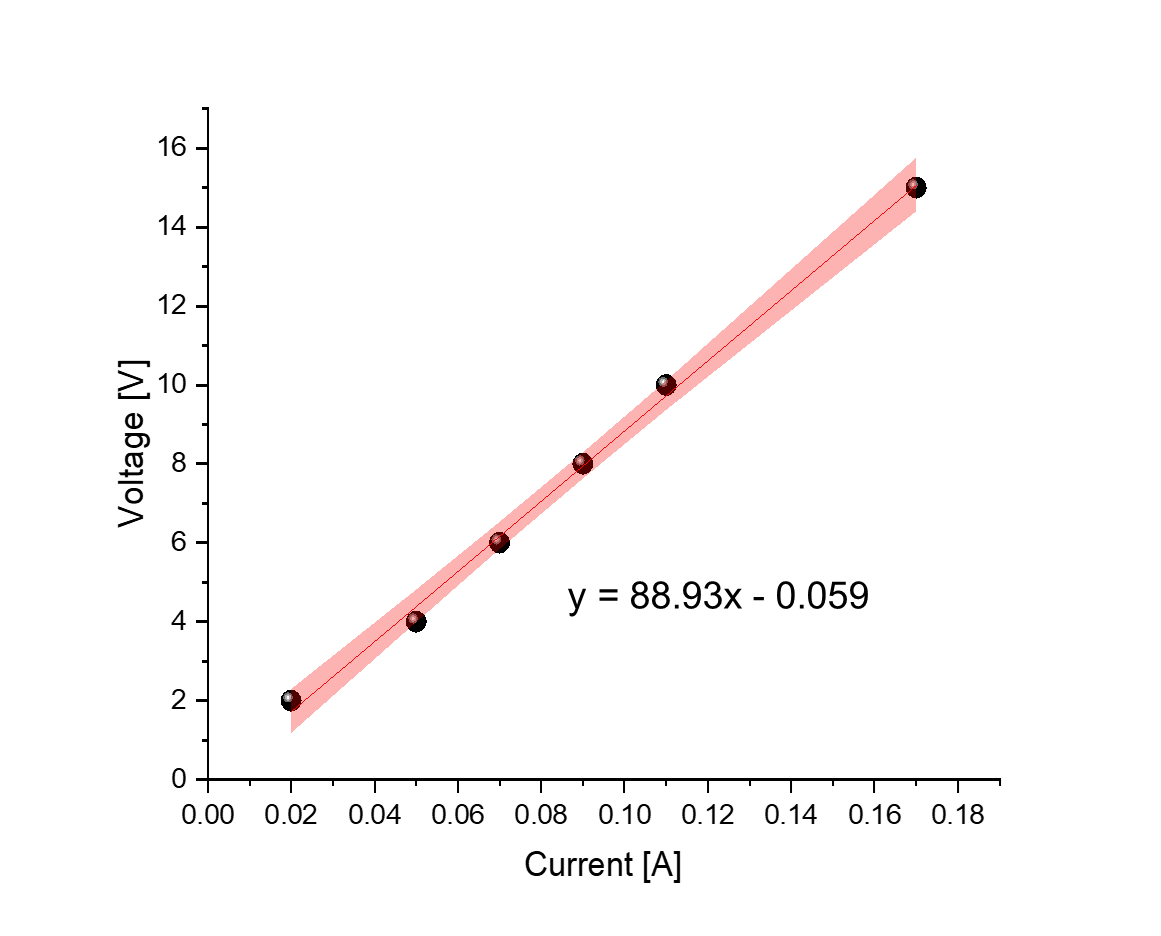


**Figure S20**. I-V characteristics of 40 nm Pt-coated polyimide film for use in Joule heating

The fabricated Pt-coated polyimide film requires a custom-built housing to obtain a stable electrical connection. We placed the capillary gripper with a Pt-coated polyimide backing between two 3D-printed plates, which were secured using bolts and nuts. All the parts produced were mounted on an automated 3-axis stage. The Joule heater exhibited stable electrical characteristics. Based on the I–V characterization, a resistance of approximately 88.9 Ω was observed within the applied voltage and current range.


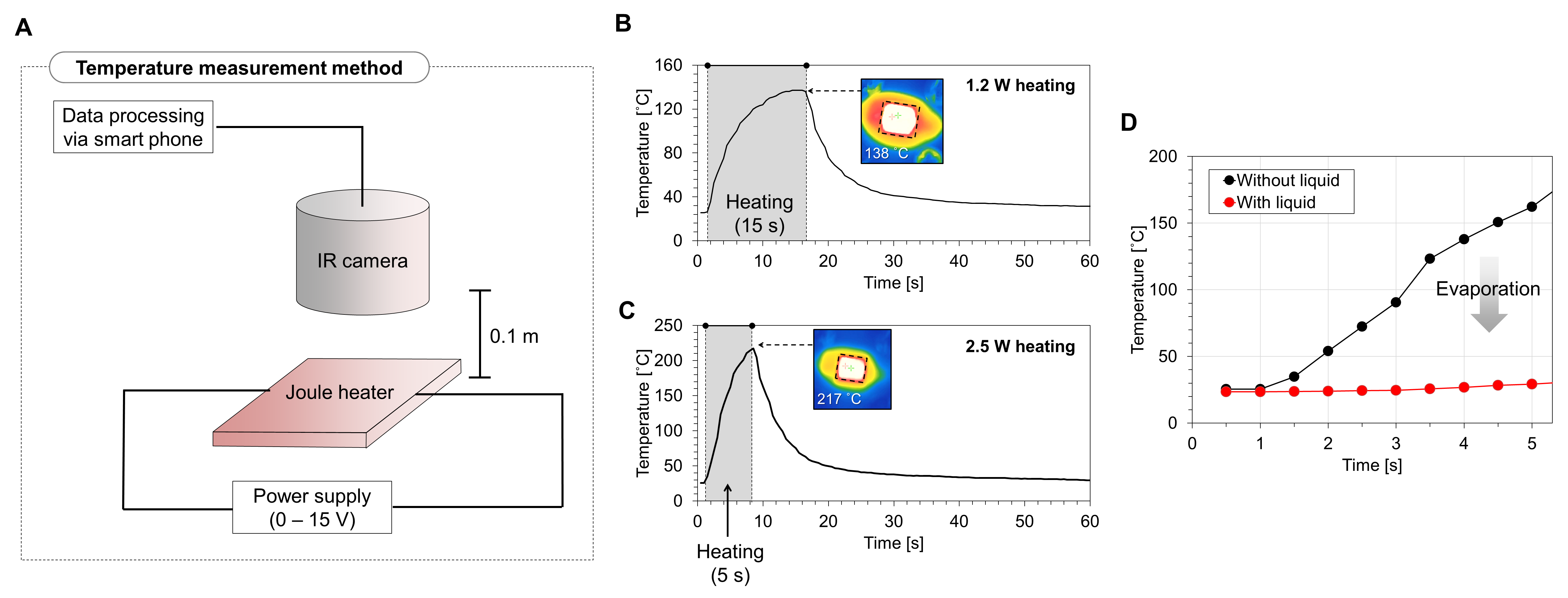


**Figure S21**. Measurement of Joule heating performance of the capillary gripper with a Pt-coated polyimide backing. (A) Schematic of surface temperature measurement using IR imaging; Surface temperature changes over time at (B) 1.2 W heating power and (C) 2.5 W heating power; (D) comparison of surface heating characteristics with and without liquid (ethanol).

We investigated the heating characteristics of the fabricated heater using an infrared imaging system (Mini 2, Hikmicro). The IR camera was placed approximately 0.1 m away from the assembled Joule heater. The emissivity of the capillary gripper surface was assumed to be 0.92.

We conducted experiments at heating powers of 1.2 W and 2.5 W. The surface temperature rose to 138 ˚C in 15 seconds at 1.2 W, and to 217 ˚C in 5 seconds at 2.5 W. When liquid is present within the nanoporous membrane, the temperature increases more slowly due to heat absorption during evaporation. This behavior is advantageous for the stable operation of the capillary gripper, as the surface temperature remains below the decomposition temperature of polyimide while enabling accelerated evaporation through Joule heating.


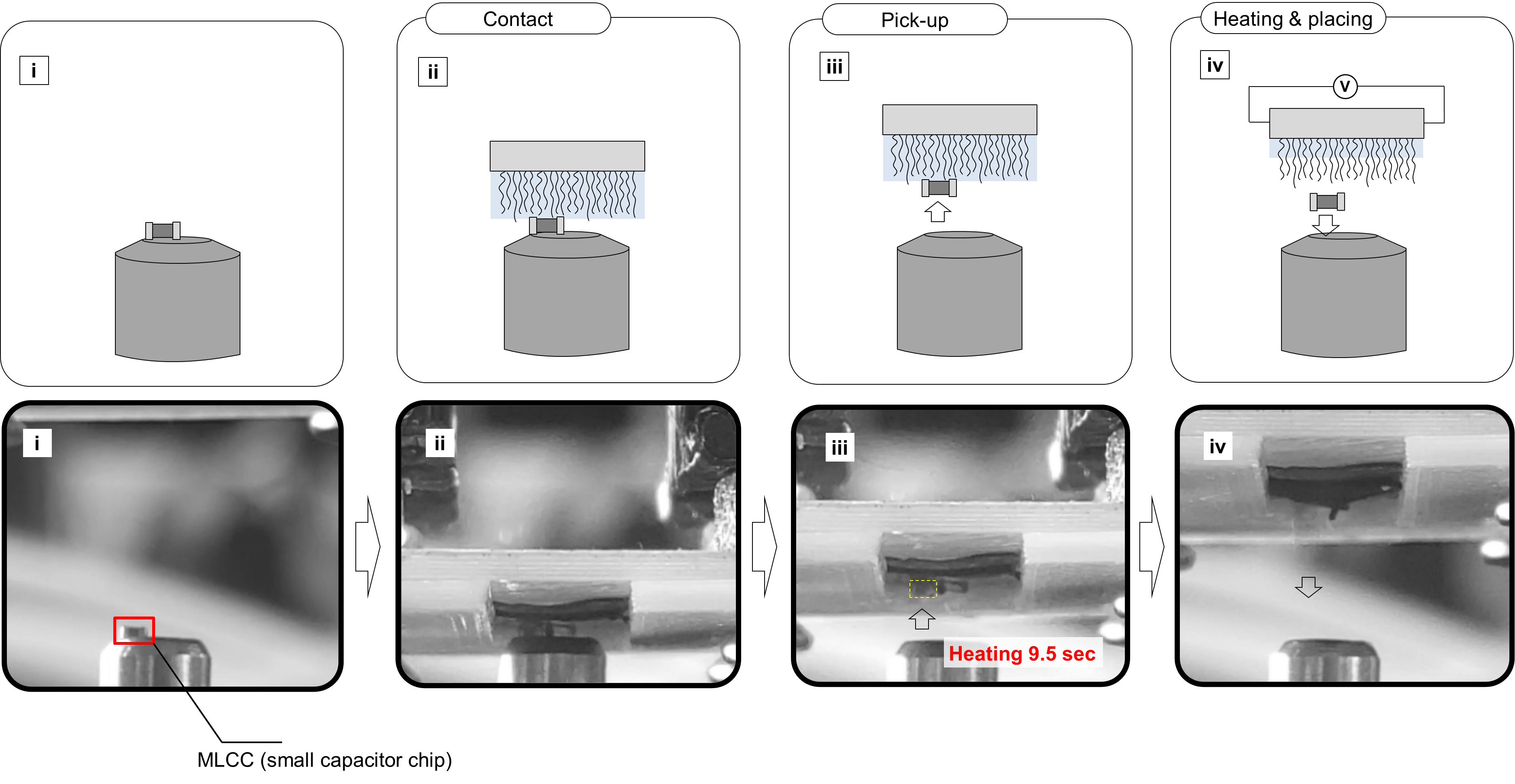


**Figure S22**. Demonstration of capillary gripping with a reduced release time
through improved heat conduction (Pt-coated polyimide).

We demonstrated that the release time could be reduced to less than 10 seconds (9.5 seconds) by applying an improved Joule heating strategy with enhanced heat conduction. This result was obtained with 20 µL of ethanol, and the adhesion switching speed can be further improved by reducing the liquid volume or changing the type of liquid.

**
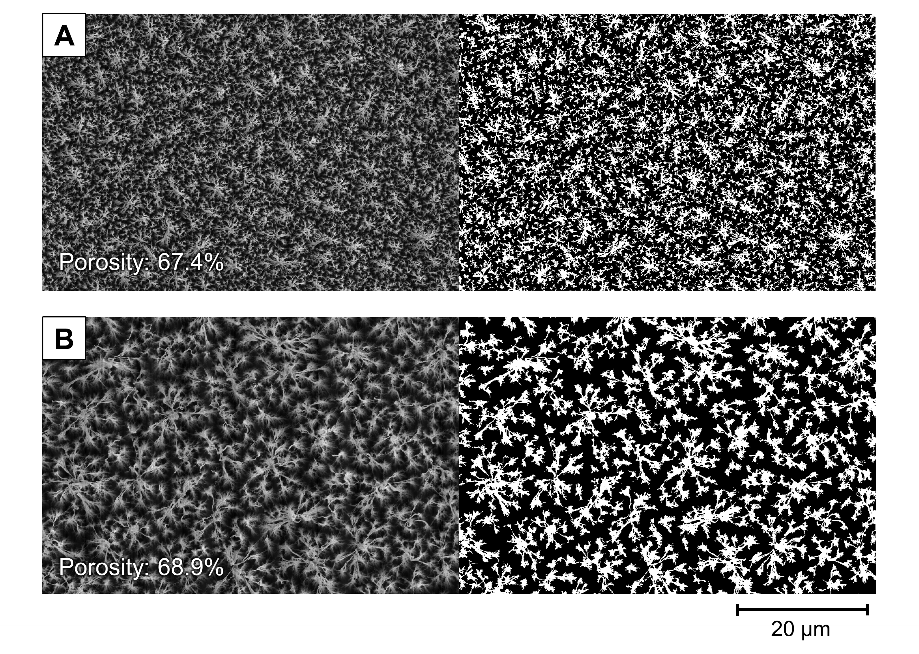
**

**Figure S23.** Surface porosity values estimated from plasma-etched surfaces
with plasma etching time of (A) 4 minutes, and (B) 8 minutes.


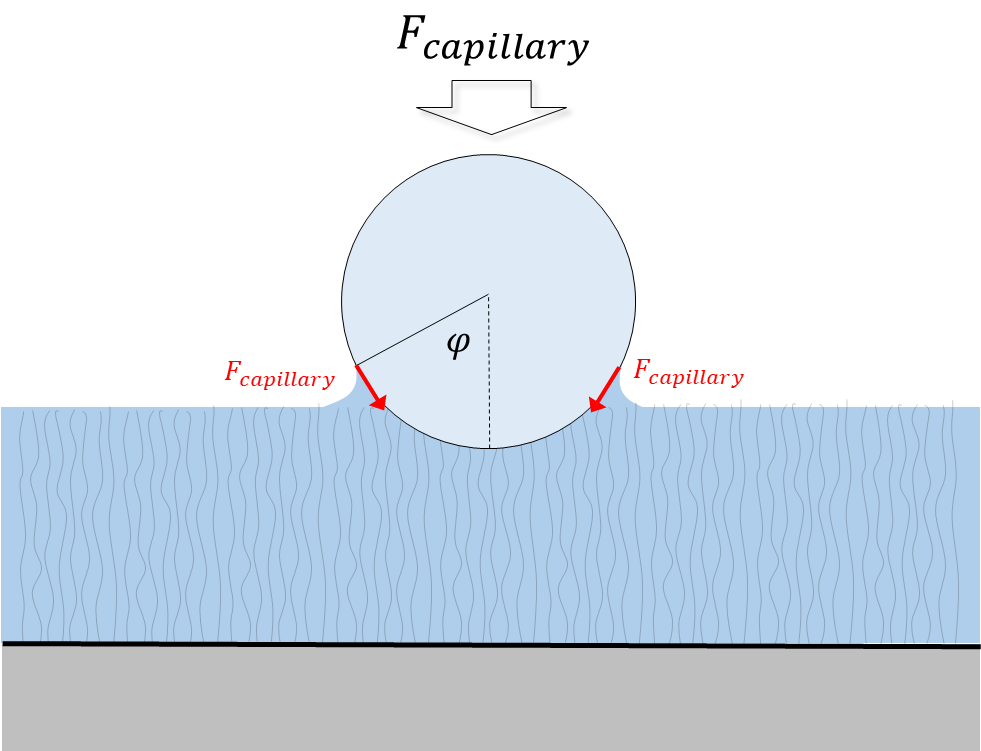


**Figure S24.** Applied force between a microsphere and a wet, nanoporous surface

The capillary force acting on the microsphere depends on the position and diameter of the contact line ($F_{capillary}\approx2\pi R\gamma{sin}^{2} \varphi$), and when *φ* has a value of 30˚, it can be expected to have a size of about 116 nN when the liquid has a surface tension of 22.4 mN m^-1^ (e.g. ethanol).

**Figure S25**. Placement error at different distances
between the receiver surface and the gripper.

**
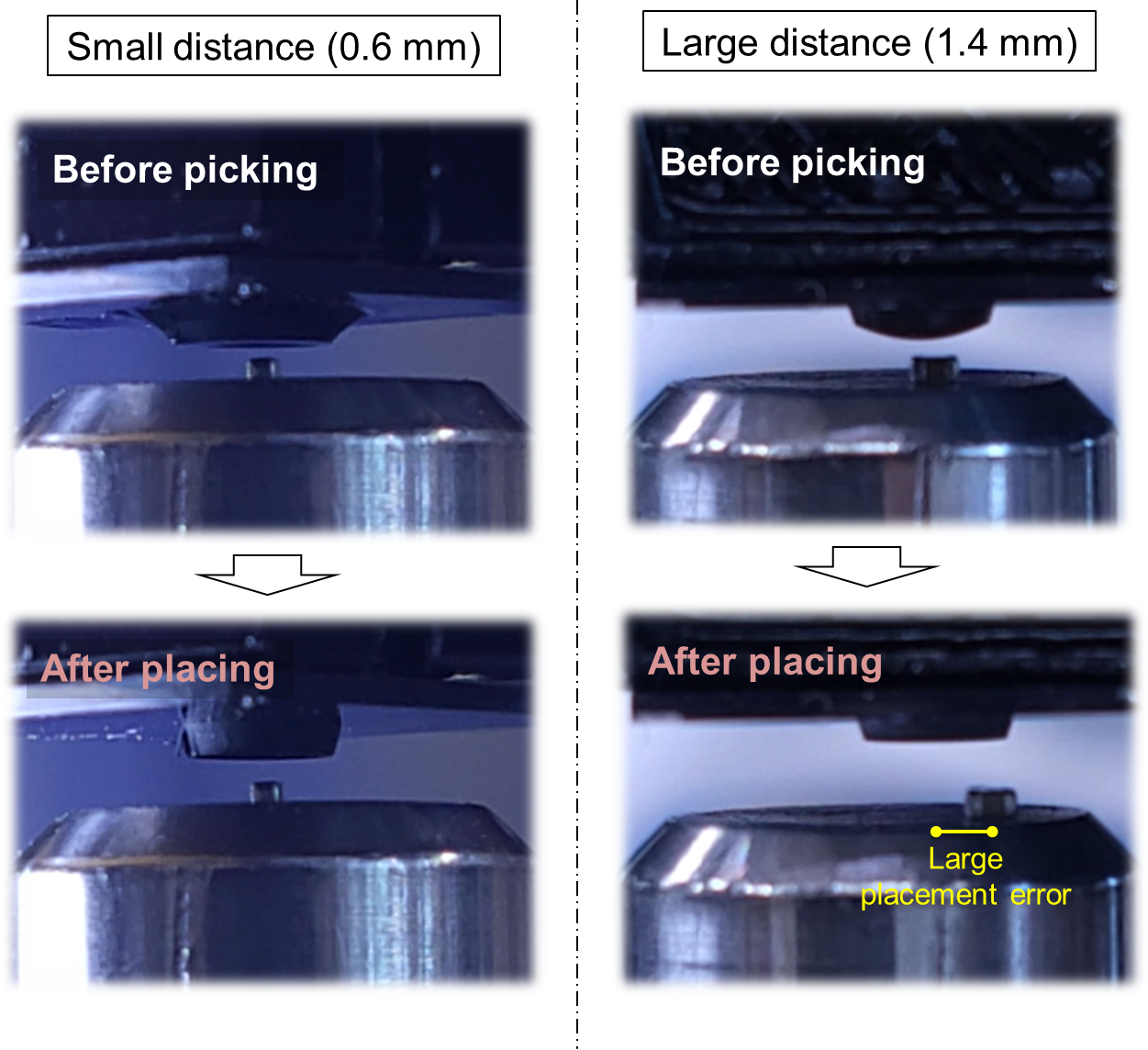
**

**Figure S26**. Photographs showing placement error at different distances between the receiving surface and the gripper.

We conducted placement experiments at separation distances of 0.6 mm, 0.9 mm, and 1.4 mm and observed that the placement error significantly decreased with decreasing separation distance. It is envisioned that with improved machine precision and alignment monitoring systems, even higher placement accuracy can be achieved than observed in the current study.

The gap between the capillary gripper and the receiving surface was controlled by a precision 3-axis stage, and the entire process from picking to placing was recorded using a camera. The experiment was repeated nine times at three different distances. Placement error values were obtained via image processing of the recorded video. Ethanol was used as the gripping liquid, and the object used for pick-and-place was a small capacitor (0.5 mm × 1 mm).

**
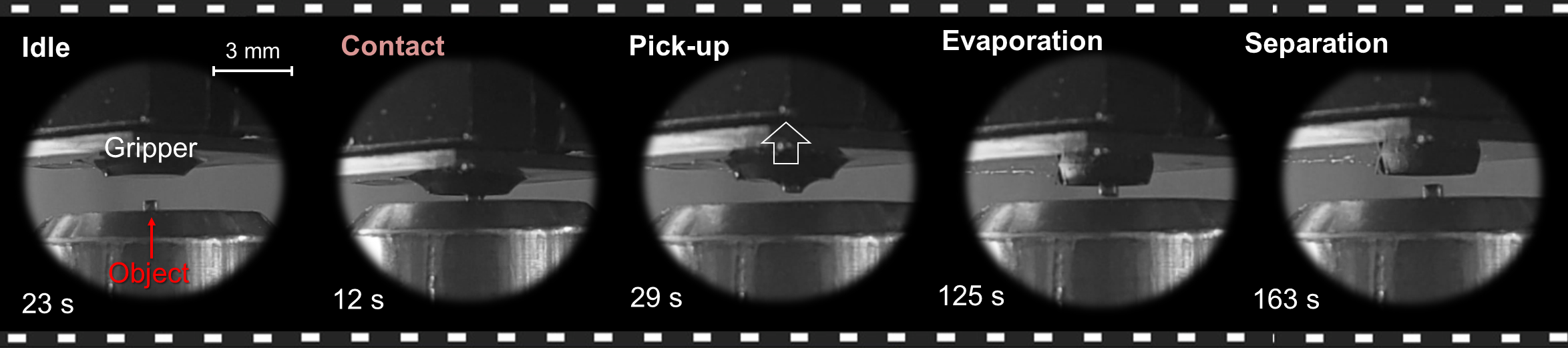
**

**Figure S27**. Snapshots of a demonstration video showing improved placement accuracy
at a close release distance.


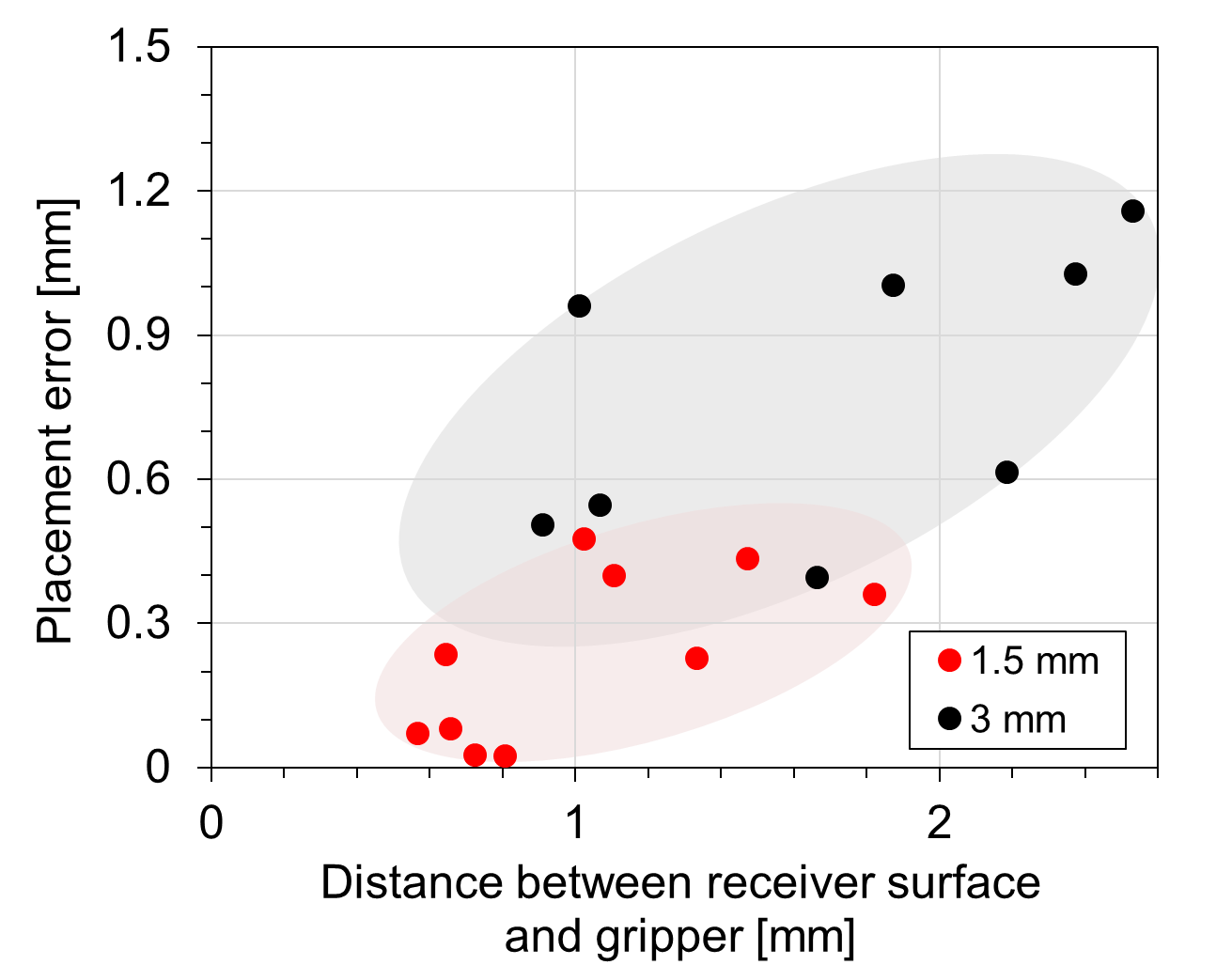


**Figure S28**. Placement error with different gripper diameters
at various separation distances.

We also performed pick-and-place demonstrations using grippers of different sizes (1.5 mm and 3 mm). A small capacitor (1 mm × 0.5 mm) was used as the target object. Experimental results showed that the placement error was lower for smaller grippers. This suggests that drifting (object movement caused by instability during liquid-object contact) during the picking process is more likely to occur with larger grippers.


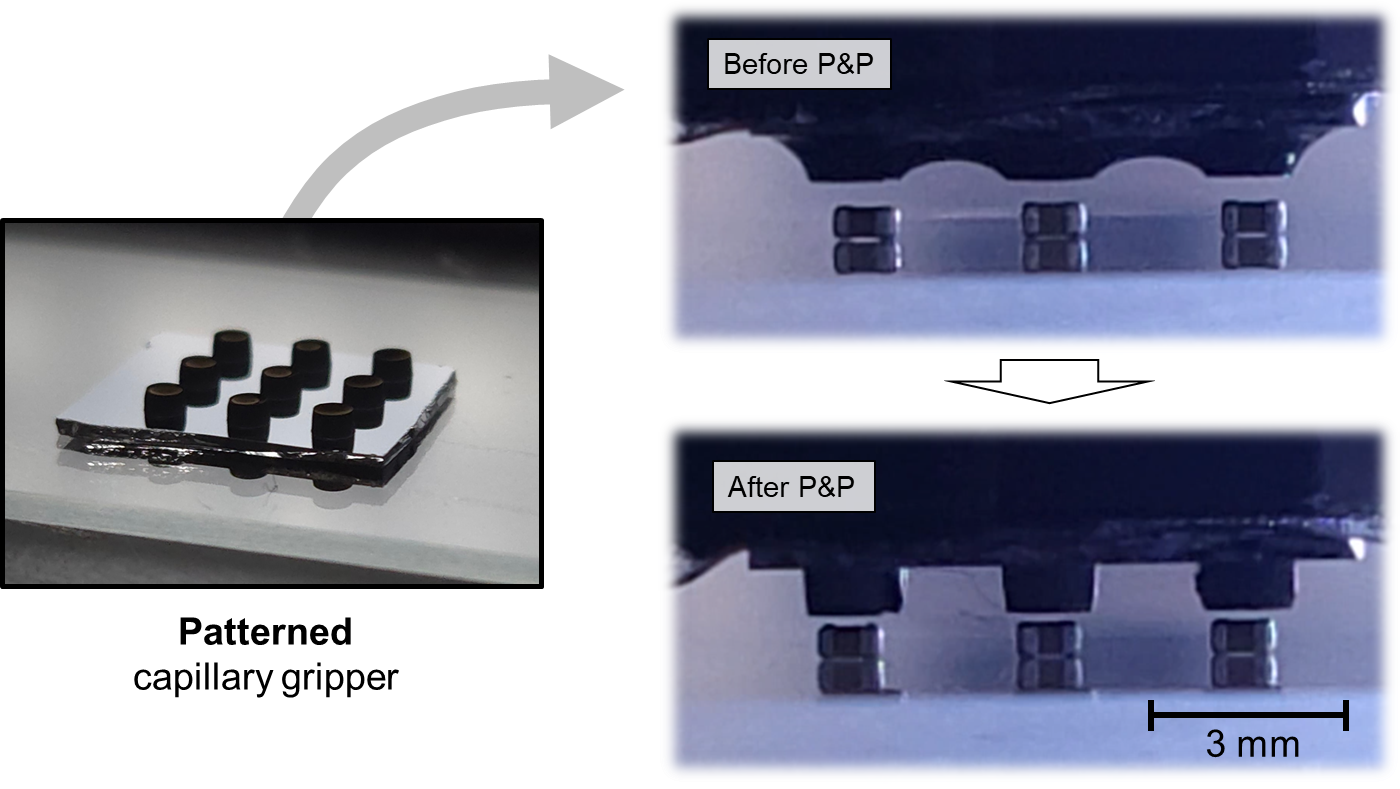


**Figure S29**. Simultaneous pick-and-place of multiple objects

using a patterned capillary gripper


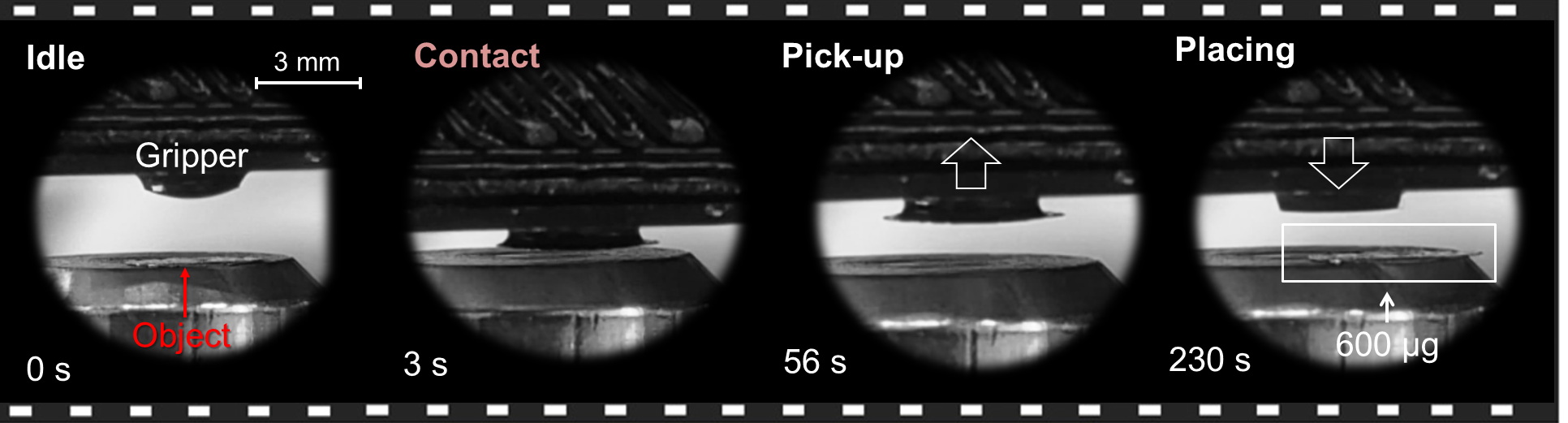


**Figure S30**. Video snapshots of solid-contactless release of
0.6 mg aluminum foil during the 20th pick-and-place cycles.


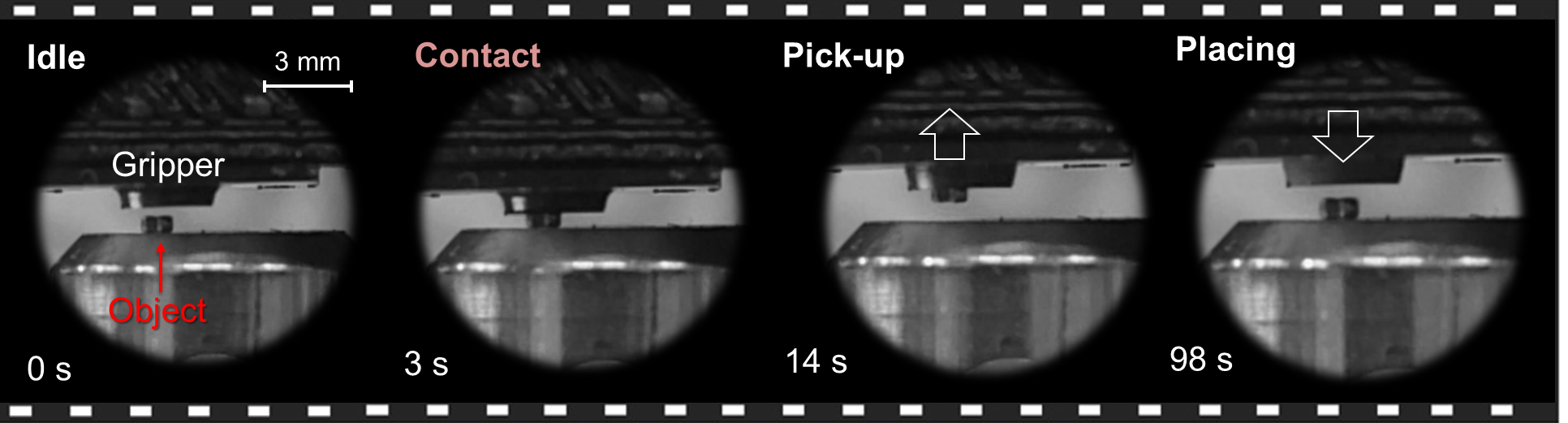


**Figure S31**. Video snapshots of solid-contactless release of
1.4 mg small capacitor chip during the 45th pick-and-place cycles.


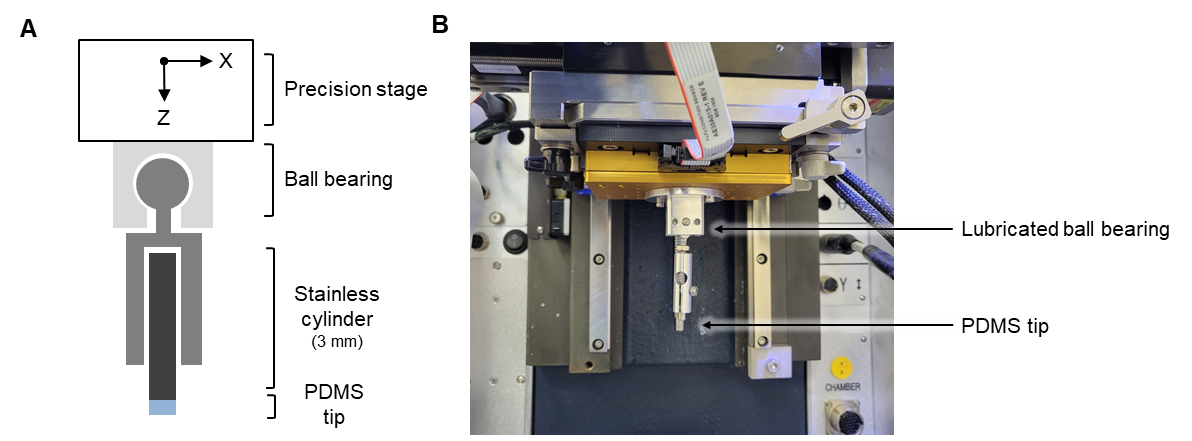


**Figure S32.** Configuration for a pull-off force measurement between PDMS and gripper surface. (A) Schematic of a jig for the testing, (B) photograph showing the test jig.

**Table S1. Geometry of thin film inductors**

| Sample number | Diameter [mm] | Turns [ - ] | Line width [ µm ] |
| --- | --- | --- | --- |
| 1 | 3 | 12 | 25 |
| 2 | 3 | 16 | 25 |
| 3 | 5 | 38 | 25 |
| 4 | 5 | 22 | 25 |
| 5 | 5 | 30 | 25 |
| 6 | 3 | 12 | 25 |
| 7 | 3 | 16 | 25 |

**Table S2. Recipe for chemical vapor deposition of vertically-aligned CNTs**

| Steps | Time  [ min ] | Wet He  [ sccm ] | Dry He  [ sccm ] | C_2_H_4_  [ sccm ] | H_2_  [ sccm ] | Temperature [ ˚C ] |
| --- | --- | --- | --- | --- | --- | --- |
| 1 | 2 | 0 | 500 | 0 | 0 | 25 |
| 2 | 3 | 10 | 500 | 100 | 400 | 25 |
| 3 | 5 | 0 | 500 | 0 | 0 | 25 |
| 4 | 5 | 0 | 100 | 0 | 400 | 25 |
| 5 | 20 | 0 | 100 | 0 | 400 | 775 |
| 6 | 5 | 0 | 100 | 0 | 400 | 775 |
| 7 | 10 | 0 | 100 | 0 | 400 | 775 |
| 8 | 1 | 0 | 100 | 0 | 400 | 775 |
| 9 | 7 | 10 | 400 | 100 | 100 | 775 |
| 10 | hydrocarbon exposure step | 10 | 400 | 100 | 100 | 775 |
| 11 | 1 | 10 | 400 | 100 | 100 | 25 |
| 12 | 10 | 0 | 500 | 0 | 0 | 25 |

**Table S3. Previous reports on active control of adhesion**

| Proposed mechanism | Materials | Minimum adhesion [kPa] | Adhesion contrast  (On-state F_ad_/Off-state F_ad_) | Reference |
| --- | --- | --- | --- | --- |
| Contact area change | Shape memory polymer | >0.9 kPa (*nominal pressure) | 1954 | *Science Advances*, 6(7), eaay5120, 2020 |
|  |  | 0.8 kPa | 746.9 - 833.4 | *ACS Applied Materials & Interfaces,* 16(7), 9443-9452, 2024 |
|  |  | 77.0 kPa | 9.34 | *Advanced Functional Materials*, 23(30), 3813-3823, 2013 |
|  |  | 9.58 kPa | 29 | *Small*, 15(50), 1904248, 2019 |
|  | Hydrogel  swelling/dehydration | 0.3 kPa | 640 | *Advanced Functional Materials*, 28(18), 1706498, 2018 |
|  | PDMS/iron NPs | 0.2 kPa | 104 | *Soft Matter*, 15(1), 30-37, 2019 |
|  | PDMS (octopus inspired adhesion) | 0.32 kPa | 293 | *Adv. Mater*, 28(34), 7457-7465, 2016 |
|  | Acrylate-based adhesive | 11 kPa | 117.5 | *npj Flexible Electronics*, 6(1), 44, 2022 |
| Interfacial chemistry | Ionogel | 5.0 kPa | 4.9 | *Advanced Intelligent Systems*, 6(2), 2300127, 2024 |
|  | Azobenzene-based  pressure-sensitive  adhesive | 4 kPa | 50 | ACS Applied Materials & Interfaces, 13(36), 43364-43373, 2021 |
| Compliance change | Phase-changing polymer | 0.5 kPa | 1989 | *ACS nano*, 18(35), 23968-23978, 2024 |
|  | Liquid metal droplet | 0.5 kPa (Si) | 224 | *Nature Communications*, 15(1), 8839, 2024 |
| External force application | Electrostatic force | 0.029 kPa | 700 | *ACS Applied Materials & Interfaces,*13(1), 1192-1203, 2020 |
| Rate-dependent adhesion | Viscoelastic elastomer (PDMS) | 75 kPa | 1.6 | *International Journal of Solids and Structures,*193, 134-140, 2020 |

Supplementary Text

Modeling of the adhesion force from the capillary gripper

The attractive force from a liquid bridge (*F_a_*) can be described as the summation of Laplace pressure force, surface tension force, and viscous force ($F_{v}$) as

$F_{a}\left( H,r \right)\approx-\Delta P\pi r^{2}+2\pi\gamma rsin\theta_{1}+F_{v}\left( \dot{H} \right)$ (S1)

, where $\Delta P$ is the pressure difference across the liquid interface, *r* is the radius of the gripper, $\gamma$ is the liquid-air surface tension, *H* is the height of the liquid bridge, $\theta_{1}$ is the contact angle on the gripper surface, and $\theta_{2}$ is the contact angle on the Si surface (Figure S33). If we assume that a retraction speed is small, the viscous term can be neglected ($F_{v}\approx0$). The distortion of the meniscus due to gravity is also negligible when the volume of the liquid bridge is small.

The pressure difference term ($\Delta P$) is derived using a circular and symmetric approximation of the liquid bridge shape based on the Young–Laplace equation as

$\Delta P=-\gamma\left( \frac{1}{R_{1}}+\frac{1}{R_{2}} \right)\approx-\gamma(\frac{cos\theta_{1}+cos\theta_{2}}{H})$ (S2)

, where *R_1_* and *R_2_* are the principal radiuses. Here, we considered the case where the gap between the two surfaces is small ($R_{1}\gg R_{2}$). Accordingly, the approximated adhesion force is written below.

$F_{a}\left( H,r \right)\approx\gamma\frac{cos\theta_{1}+cos\theta_{2}}{H}\pi r^{2}+2\pi\gamma rsin\theta_{1}$ (S3)

The volume of the liquid bridge (*V*) is approximately estimated as

$V\approx\pi r^{2}H$ (S4)

Finally, the adhesion force is expressed in terms of volume as

$F_{a}= \pi^{2}\gamma\left( cos\theta_{1}+cos\theta_{2} \right)\frac{r^{4}}{V}+2\pi\gamma rsin\theta_{1}$ (S5)

We used the values of 26.6 mN m^-1^ and 0.75 mm for $\gamma$ and *r*, respectively. $\theta_{1}$ and $\theta_{2}$ were assumed as 90˚ and 30˚ to best explain the experimental data. To estimate the liquid bridge volume ($V\approx\pi r^{2}H$), *H* value with the maximum adhesion was chosen.

Contact mechanical modeling of the gripper surface

Classical contact mechanical framework considers a surface, which is a collection of asperities with random heights following a specific probability density function. For example, the Gaussian distribution of height ($\phi$) is written as

$\phi(l)=\frac{1}{\sigma\sqrt{2\pi}}exp(-\frac{l^{2}}{2\sigma^{2}})$ (S6)

, where $l$ is the height of the asperity, $\sigma$ is the standard deviation of the height of the asperities.

When we assumed a Gaussian probability density function, the probability of contact ($P_{contact}$) of asperities can be written as

$P_{contact}=\frac{1}{2}\{1-erf(\frac{d}{\sqrt{2}\sigma})\}$ (S7)

, where *d* is the distance between the nominal contact surface of the gripper and the indenter surface.

Also, the preload pressure (*p*) is estimated from the summation of contact forces ($P_{CNT, i}$) from each nanowire as

$p=\frac{1}{A}\sum_{1}^{n_{c}} P_{CNT, i}$ (S8)

, where *A* is the nominal contact area, and *n_c_* is the total number of asperities in contact.

If we assume that the load (*P_cnt_*) - displacement ($\delta_{CNT}$) behavior of a nanowire contact is linear, the preload pressure can be represented again as

$p=\frac{1}{A}\sum_{i=1}^{n_{c}} k_{CNT}\delta_{CNT,i}=\frac{n}{A}\int_{d}^{\infty} k_{CNT}(l_{CNT}-d)\phi{dl}_{CNT}$ (S9)

After the integration of Eq. S9, *p* is again written as

$p=\frac{k_{CNT}\sigma\varphi}{\sqrt{2\pi}}[\exp\left( -\frac{d^{2}}{2\sigma^{2}} \right)-\sqrt{\frac{\pi}{2}}\frac{d}{\sigma}\{1-erf \frac{d}{\sqrt{2}\sigma}\}]$ (S10)

, where erf() is the error function of which definition is $\mathrm{erf} \left( x \right)=\frac{2}{\sqrt{\pi}}\int_{0}^{x} e^{-t^{2}}dt$, and $\varphi$ is the number density of the nanowires.

Similarly, pull-off pressure ($P_{pulloff}$) can be written as

$P_{pulloff}=\varphi P_{CNT}\frac{1}{2}\{1-erf(\frac{d}{\sqrt{2}\sigma})\}$ (S11)

When the surface is not plane as shown in the Figure S34, we use an area integration to estimate the total force as

$F_{preload}=\int pdA=\int2\pi r\frac{k_{CNT}\sigma\varphi}{\sqrt{2\pi}}[\exp\left( -\frac{d^{2}}{2\sigma^{2}} \right)-\sqrt{\frac{\pi}{2}}\frac{d}{\sigma}\{1-erf \frac{d}{\sqrt{2}\sigma}\}]dr$ (S12)

$F_{pull-off}=\int P_{pulloff}dA=\int2\pi r\varphi P_{CNT}\frac{1}{2}\{1-erf(\frac{d}{\sqrt{2}\sigma})\}dr$ (S13)

From the geometrical consideration, the relation between *d* and *r* can be written as

$d\left( r \right)=D+R-\sqrt{R^{2}-r^{2}}$ (S14)

, where *D* is the distance between the tip of the spherical indenter and the nominal contact surface, and *R* is the radius of the indenter.

For the modeling, we used the values of $\varphi$, $\sigma$, $k_{CNT}$, $a_{CNT}$, and *R* of 10 µm^-2^, 0.14 µm, 3 N m^-1^, 0.01 µN, and 3.31 µm, respectively.


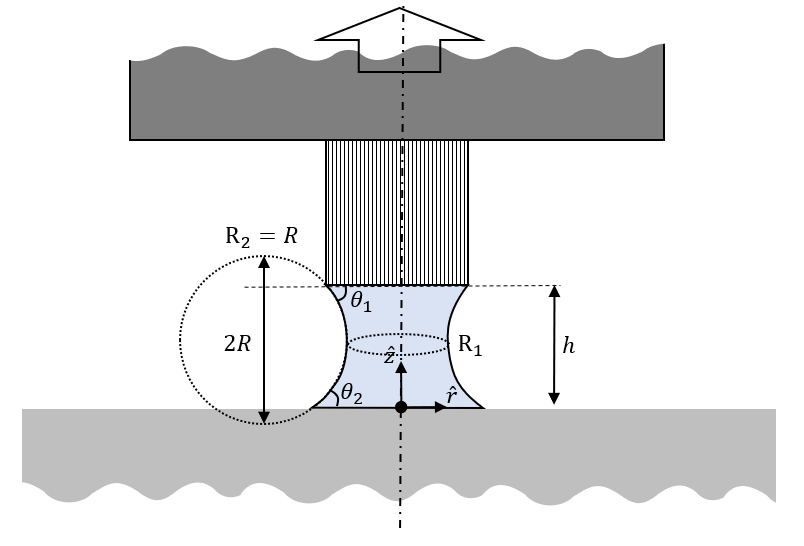


**Figure S33.** Configuration used in the adhesion modeling of the capillary gripper


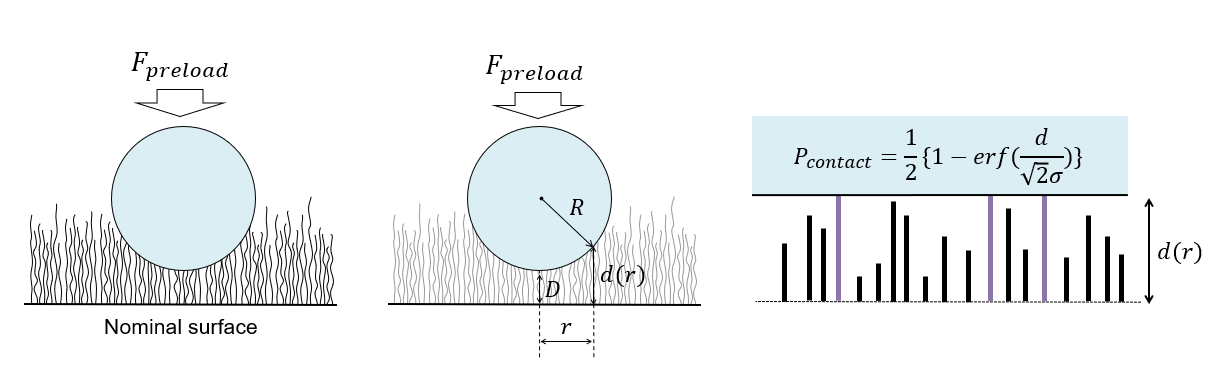


**Figure S34.** Configuration used in the nanocontact mechanics modeling of the capillary gripper

**Movie S1.**

Video showing solid-contactless placing of light objects using a capillary gripper with polyimide backing.

**Movie S2.**

Picking and placing a thin, lightweight polymer sheet using a capillary gripper

**Movie S3.**

Video showing limited elastocapillary densification of the capillary gripper structure during ethanol evaporation.

**Movie S4.**

Picking and placing small LED elements using an engineered capillary gripper surface

**Movie S5.**

Surface temperature during Joule heating of a capillary gripper with a Pt-coated polyimide backing

**Movie S6.**

Picking and placing a micro-architectured material with very small pressures

**Movie S7.**

Video showing the process of integrating LEDs onto micro-architectured materials

**Movie S8.**

Precise solid-contactless placement through the minimized distance between the receiver surface and the gripper

**Movie S9.**

Simultaneous pick-and-place of multiple objects using a patterned capillary gripper

**Movie S10.**

Successful 45 repetitive pick-and-place cycles without placing failure
